# Aerodynamic Performance of a Baja SAE Vehicle Using Hybrid RANS-LES Approach

**DOI:** 10.12688/f1000research.171076.1

**Published:** 2025-11-03

**Authors:** Sergio A. Ardila Gomez, Jessica G. Maradey Lazaro, Jhon J. Quiñones, Deisy Y. Rodriguez Sarmiento

**Affiliations:** 1 Universidad Autonoma de Bucaramanga, Bucaramanga, Santander, Colombia; 2 Purdue University, West Lafayette, Indiana, USA

**Keywords:** Baja SAE vehicle aerodynamics, Hybrid RANS LES turbulence modeling, Computational Fluid Dynamics CFD, Drag and lift coefficients, Flow separation and wake dynamics

## Abstract

This study presents a high-fidelity computational investigation of the aerodynamic performance of a Baja SAE off-road vehicle using a hybrid Reynolds-Averaged Navier–Stokes (RANS) and Large Eddy Simulation (LES) turbulence modeling approach. The methodology combines the Spalart–Allmaras RANS model for near-wall flow treatment with Detached Eddy Simulation (DES) for resolving large-scale unsteady turbulent structures in the vehicle’s wake. A detailed computational domain and refined meshing strategy were implemented using ANSYS Fluent, including mesh adaptation based on the LES filter size (Δ = 23.5 mm) and mesh independence validation.

Simulations were performed under steady (RANS) and unsteady (DES) conditions at 30 km/h, yielding a drag coefficient (Cd) of 1.290 for RANS and 1.249 for DES. While RANS provided stable results with low variance (σ = 0.005), the DES model captured transient phenomena such as vortex shedding and near-wake recirculation with higher accuracy (σ = 0.024), enhancing the prediction of flow separation zones and aerodynamic forces. Pressure and velocity field analyses revealed improved resolution of stagnation zones and vortex dynamics under the DES framework, particularly around the roof, rear, and underbody regions.

The Q-criterion visualization showed that DES allowed the identification of both large-scale and fine-scale vortex structures in the near and far wake, offering a comprehensive representation of turbulence intensity and flow instabilities. These findings confirm the suitability of hybrid RANS–LES methods for aerodynamic optimization of complex vehicle geometries, providing enhanced predictive capabilities compared to traditional steady-state models.

## 1. Introduction

The Baja SAE competition is an international engineering challenge that promotes the design, analysis, and manufacturing of off-road vehicles by university students under real-world constraints. Participating teams must address key aspects such as safety, reliability, regulatory compliance, cost-efficiency, and overall performance. ^1^ Beyond technical development, the project fosters management and teamwork skills, aligning with educational objectives outlined by the Accreditation Board for Engineering and Technology (ABET). ^2, 3^

The vehicle must meet critical requirements including robustness, ease of maintenance, driver accessibility, and the ability to traverse rough terrain—all within a limited budget of $2,500. ^4^ To achieve optimal performance, technical efforts focus on chassis design, material selection, and the improvement of mechanical systems such as suspension and steering. ^1^ Structural integrity is assessed using Finite Element Analysis (FEA), which enables virtual evaluation of stress, deformation, and vibration modes under various loading scenarios typical in Baja SAE events, such as acceleration, endurance, and impact tests. ^4, 5^ Static and dynamic analyses help prevent structural failure and enhance occupant safety, while software tools like ANSYS, COMSOL, or LS-DYNA are used for advanced simulations including frontal crashworthiness and torsional stiffness validation. ^6, 7^

In addition to structural strength, aerodynamic performance plays a key role in reducing drag, improving stability, and optimizing fuel efficiency. Aerodynamic assessments can be conducted experimentally or numerically, with Computational Fluid Dynamics (CFD) emerging as a cost-effective alternative to wind tunnel or on-road testing. ^8, 9^ CFD allows accurate prediction of flow behaviour including drag, lift, vortex shedding, and pressure distributions using only the vehicle’s 3D geometry. This methodology accelerates the design process and reveals flow inefficiencies that may be overlooked through conventional design approaches. ^10, 11^ Drag force is a major contributor to fuel consumption at high speeds, ^8^ while lift affects tire adherence and vehicle stability. Understanding these parameters is essential for performance optimisation. Table 1 summarise recent studies involving FEA and CFD in the design of Baja SAE vehicles.

**Table 1.** Overview of relevant studies involving Finite Element Analysis (FEA) and Computational Fluid Dynamics (CFD) applied to structural and aerodynamic optimisation of Baja SAE vehicles.

Reference & year	Methods used	Key findings
Zhang et al., 2024 ^12^	Vehicle Dynamics Modeling, sensor instrumentation	Validated vehicle dynamics, improving prototype design
Franco-Camacho et al., 2020 ^13^	Multi-body dynamics, FEA for suspension & chassis	Optimized suspension and chassis, validated handling & strength
Zainal et al, 2018 ^14^	FEA structural & modal analysis with experimental validation	Optimized resonance modes, improving structural safety
Bhardwaj et al., 2023 ^15^	FEA design & optimization of steering assembly	47% weight reduction, enhanced handling
Vinod et al, 2017 ^16^	Modal Analysis of chassis with CAD and ANSYS	Identified natural frequencies, improved durability
Khan et al., 2023 ^17^	CAD and power transmission simulation for driveshaft	Optimized driveshaft design for off-road 4x4 system
Kumar et al., 2024 ^18^	CAD and FEA design of customized brake caliper	Created brake caliper supporting 315,645 Nm torque
Maradey et al., 2019 ^1^	CFD Simulations (ANSYS Fluent) for aerodynamics analysis	Improved airflow understanding, drag, lift, pressure distribution
Silva et al., 2023 ^19^	Suspension design and MSC Adams Car© (with aerodynamic considerations)	Double-stage springs improved dynamic response and aerodynamics
Trinadh Raju et al.,2025 ^20^	CFD (ANSYS) for aerodynamics; FEA for impact analysis	Drag force 185N at 60km/h; validated structural integrity
Abu Farjad & Predators Racing, 2025 ^21^	CFD (Ansys Fluent) and FEA for drag reduction & optimization	5% drag reduction and 6.5kg weight saving through simulation

CAD (Computer Aided Design).

Computational Fluid Dynamics (CFD) has become a vital resource in the aerodynamic development of Baja SAE vehicles, enabling accurate analysis of complex flow phenomena—such as drag, lift, vortex shedding, and pressure distribution—using only the vehicle’s 3D geometry. By eliminating the need for physical prototypes, CFD accelerates design iterations, reduces costs, and enhances precision in flow prediction. It supports the optimisation of key components like bodywork, intakes, and cooling systems, improving thermal performance, reducing aerodynamic drag, and increasing overall vehicle stability. Additionally, CFD visualisations of pressure and velocity fields provide critical insights during early development, making it a cost-effective and powerful alternative to experimental methods like wind tunnel or road testing ^1, 22– 24^ The general CFD workflow followed in this study is summarised in Figure S1 (see Supplementary Material 1).

To simulate these flow phenomena with sufficient accuracy and computational efficiency, particularly in regions with flow separation and vortex dynamics, the selection of an appropriate turbulence model is essential. Turbulent flows pose significant challenges due to their inherent unsteadiness and wide range of scales. While Direct Numerical Simulation (DNS) offers the most accurate solution, it is computationally prohibitive for full-vehicle analysis. Therefore, turbulence modeling strategies such as Reynolds-Averaged Navier–Stokes (RANS), Large Eddy Simulation (LES), and hybrid RANS–LES frameworks are widely used. RANS is commonly applied near walls for its efficiency, while LES resolves large-scale structures in regions such as wakes and shear layers. ^25, 26^

Hybrid models combine both approaches, leveraging the strengths of RANS in boundary layers and LES in separated flow regions to balance accuracy and computational cost. A critical component of these models is the transition mechanism between RANS and LES zones, managed via zonal interfaces or dynamic blending functions based on local flow properties. ^25^ Among the most commonly used hybrid methods are Detached Eddy Simulation (DES), Delayed Detached Eddy Simulation (DDES), and Scale-Adaptive Simulation (SAS). DDES improves stability by preventing premature switching and reducing sensitivity to grid resolution, ^27^ while SAS dynamically adjusts turbulence length scales based on local conditions, eliminating the need for manual interface definitions. ^26^ A comparative overview of these turbulence models is provided in Figure S2 (see Supplementary Material 2).

This article outlines the aerodynamic analysis methodology for a Baja SAE vehicle. Section 2 describes the geometry, meshing strategy, RANS and DES turbulence models, and simulation setup. Section 3 presents results comparing both models in terms of drag prediction, flow fields, vorticity, and 3D vortex structures.

## 2. Methods

### 2.1 Geometry and computational domain

**Figure 1.**
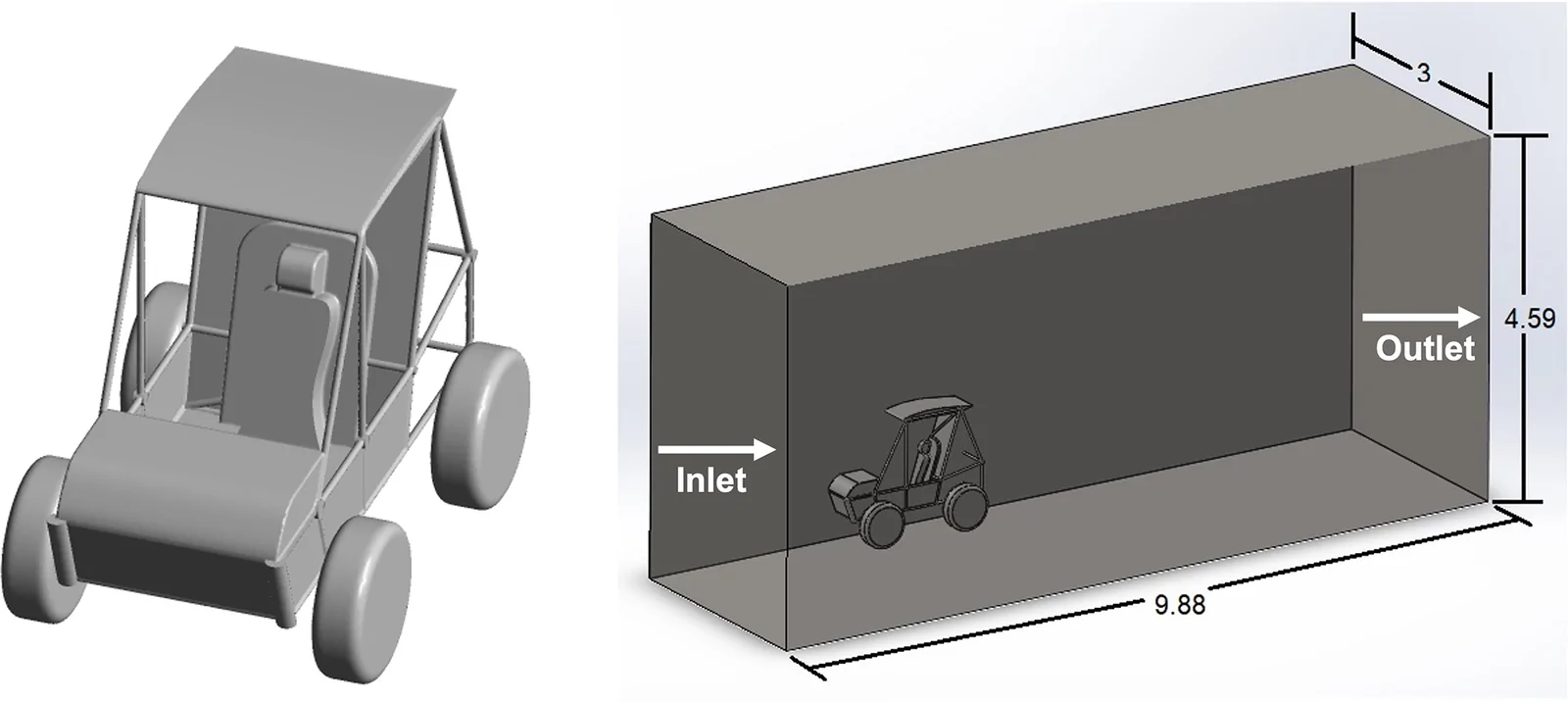
Vehicle geometry and computational domain.

The 3D model of the Baja SAE vehicle used in this study is shown in Figure 1a. It was developed based on the geometric specifications listed in Table 2. To reduce computational cost, several geometric details—particularly in the engine bay, underfloor region, and driver compartment—were simplified. Additionally, aerodynamic refinements were implemented on the vehicle’s front and roof areas to improve flow behaviour and streamline continuity.

**Table 2.** Vehicle and computational domain dimensions.

Baja SAE		Computational domain	
Dimension	Value	Dimension	Value
**Height**	1530 mm	Model incident flow	1.3 x vehicle length
**Width without wheels**	856.8 mm	Model – outflow	3 x vehicle length
**Total length**	1883.71 mm	Total width	7 x vehicle width
**Front area (half )**	0.60033 m ^2^	Model – roof	3 x vehicle height
**Characteristic length (between axis)**	1175.85 mm		

The computational domain dimensions are shown in Figure 1b and summarized in Table 2, and were selected following established guidelines from previous aerodynamic simulations to avoid influence from far-field recirculation and ensure numerical accuracy. ^28, 29^ A symmetry boundary condition was applied along the lateral plane, which effectively halves the simulation domain when the flow is symmetric, significantly reducing computation time without compromising fidelity ^30^

A) 3D CAD model of the Baja SAE vehicle; B) Schematic of the computational domain with dimensions in meters.

### 2.2 Meshing

**Figure 2.**
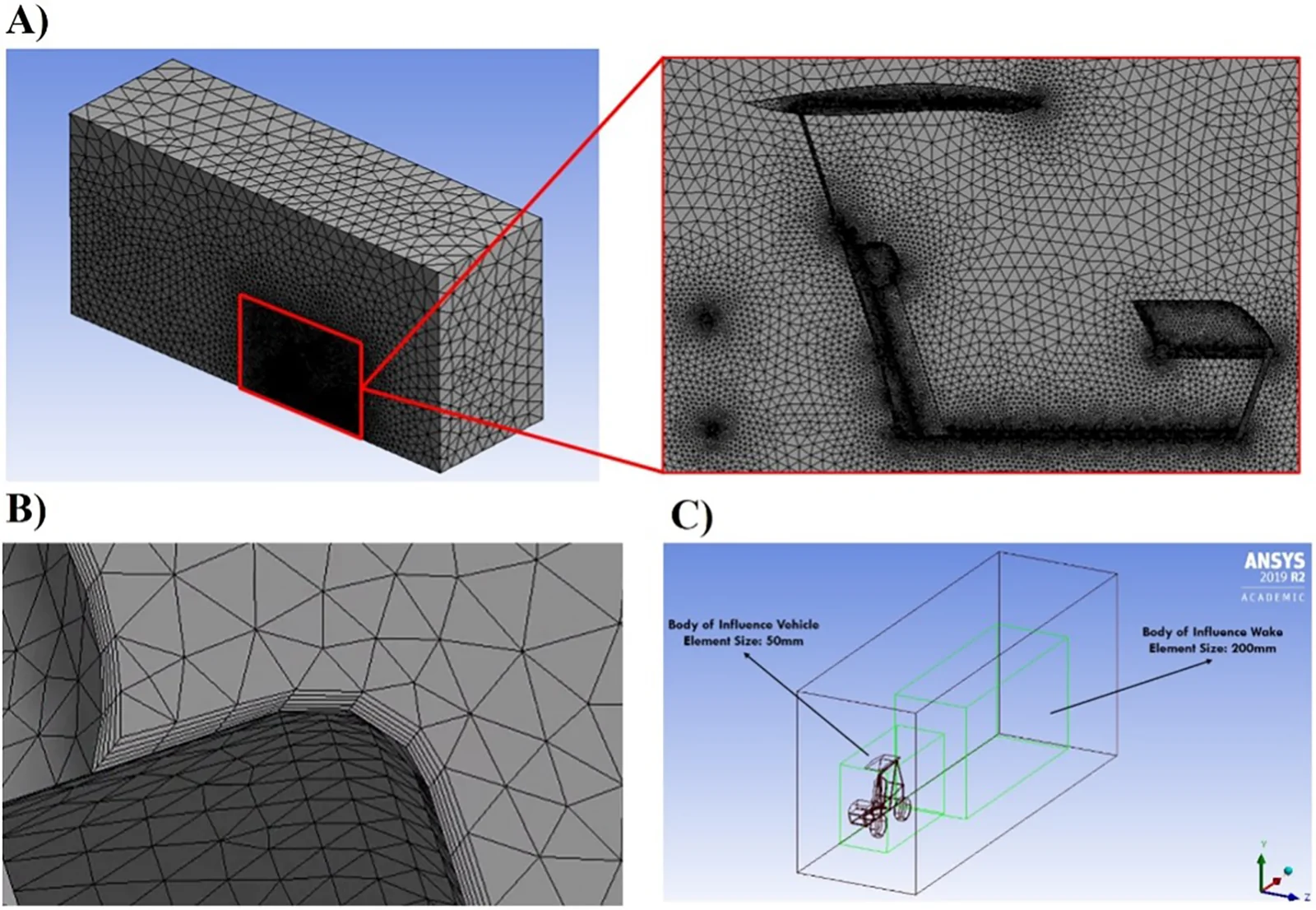
A) Computational domain and vehicle mesh; B) Detail of the prism layer on the vehicle; C) Body of influence boxes.

Figure 2a shows the hybrid mesh generated in the ANSYS meshing module. This mesh consists of tetrahedral elements throughout the domain and prism layers near the vehicle surface, where the turbulent boundary layer is expected ( Figure 2b). The prism layer was configured with an initial height of 0.393 mm, a growth rate of 1.12, and five total layers, resulting in a non-dimensional wall distance (Y ^+^) below 5. Prism elements were also applied to the tire surfaces.

To improve resolution around the vehicle and its wake, two box-shaped bodies of influence were added ( Figure 2c), using element sizes of 50 mm near the vehicle and 200 mm in the wake region. These refinements help capture vortical structures at multiple scales and ensure a smooth transition from near-field to far-field mesh densities.

A mesh independence study was conducted using six mesh configurations with total cell counts ranging from 800,000 to 10 million. The drag coefficient (Cd) was used as the convergence criterion, and mesh independence was achieved with approximately 1.16 × 10 ^6^ elements. Mesh quality was evaluated using the Jacobian determinant, with most elements scoring between 0.7 and 0.95, which is acceptable for accurately resolving boundary layer phenomena. Results of the mesh independence analysis are presented in Figure S3 (see Supplementary Material 3).

### 2.3 RANS Model implementation

In the implemented model, the airflow was assumed to be incompressible (isothermal), Newtonian, and three-dimensional. The working fluid was dry air under standard conditions (T = 25°C, 1 atm), corresponding to a Reynolds number of 6.7 × 10 ^5^, based on the vehicle’s wheelbase.

The boundary conditions were defined as follows: a uniform inlet velocity of 30 km/h (8.33 m/s) was imposed at the domain entrance ( Figure 1b), while the outlet was set to constant atmospheric pressure. The lateral, upper, and symmetry planes were treated as symmetry boundaries (zero-gradient) to avoid artificially enclosing the wake region and to ensure realistic flow development. The ground, vehicle chassis, and tyres were modelled as stationary no-slip walls.

Simulations were performed using ANSYS FLUENT v19.2 ^31^ under steady-state conditions. The one-equation Spalart–Allmaras turbulence model ^32^ was employed for the RANS formulation due to its efficiency in external aerodynamic flows at high Reynolds numbers and its relatively low computational cost. The transport equation for the model is expressed as Eq 1:

$$\frac{\partial }{\mathrm{\partial t}}\left(\rho \tilde{v}\right)+\frac{\partial }{\partial x_{i}}\left(\rho \tilde{v}u_{i}\right)=G+\frac{1}{\sigma }\left\{\frac{\partial }{\partial x_{j}}\left[\left(\rho \tilde{v}+\mu \right)\;\frac{\partial \tilde{v}}{\partial x_{j}}\right]+C_{b}{\left(\frac{\partial \tilde{v}}{\partial x_{j}}\right)}^{2}\right\}-Y$$

**Equation 1.** Transport equation for the modified turbulent kinematic viscosity ν in the Spalart–Allmaras one-equation turbulence model. ^32^

where *G* represents the production of turbulent viscosity, *Y* is its destruction near the wall due to viscous damping and wall-blocking effects, *σ* and *cb* are empirical constants, and *ν* is the molecular viscosity. A second-order spatial discretisation scheme was used, and pressure–velocity coupling was achieved through the COUPLED algorithm. Convergence was considered achieved when the residuals dropped below 1 × 10 ^−5^ within 5000 iterations.

### 2.4 DES Model adaptation

**Figure 3.**
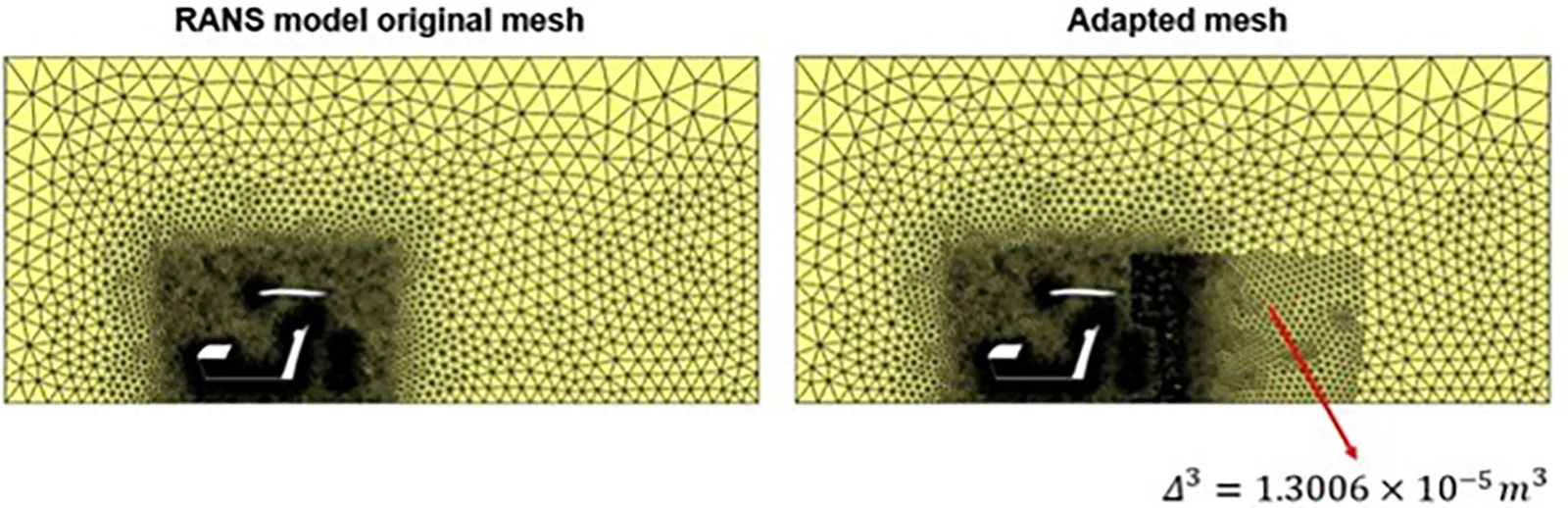
Mesh adaptation from RANS to DES.

The hybrid RANS-LES turbulence model DES was implemented in this study to obtain more accurate results and improve the visualization of the vorticity iso-surfaces in the near wake of the Baja SAE vehicle. In the DES model, the flow field can be divided into three regions: Euler Region, RANS Region and LES Region. The boundary layer regions are modelled with unsteady RANS (URANS) and the outer detached vortices are captured with LES. ^33, 34, 35^ The LES region and the called grey zones were calculated based on the guidelines reported on. ^35^ First, the LES filter was estimated to divide the characteristic length of the vehicle (distance between wheels) by 50. It results in a LES filter value of Δ = 23.517 mm, which is less than the smallest vortex diameter observed in the vorticity contours of the RANS model ∅ _min_ = 33.6 mm. The smallest vortex diameter was measured in a vorticity contour located in the near wake of the vehicle using the MATLAB algorithm developed by Smith R. ^30^ Then, the volume of refinement of the cells (∆ ^3^ = 1.3006 × 10 ^−5^ m ^3^) was calculated using the Filter LES value. Finally, the calculated volume of refinement was used to adapt the mesh in ANSYS FLUENT ( Figure 3). The total number of elements of the adapted mesh was 7 million as shown in Figure 3.

The time step for the DES simulations is calculated according to ^9^ as ∆t = ∆/Umax = ∆/U∞ = 2.822 × 10 ^−3^ s, where Umax, is the maximum velocity registered in the simulations, which in this case is the velocity of the incident fluid flow. The total time of analysis was determined as t = 4 Lcar/U∞ = 0.904s and 40 iterations per time step were used, where Lcar is the total length of car model.

## 3. Results

### 3.1 Drag coefficient

For the RANS model, the drag coefficient (Cd) was calculated by averaging the values from the final 100 iterations of the steady-state simulation. The resulting Cd was approximately 1.290, with a standard deviation of 0.005. This low deviation indicates good convergence stability of the RANS solution. In contrast, the hybrid RANS–LES model was implemented under unsteady-state conditions. The time-averaged Cd, computed from the instantaneous values at each timestep, was 1.249, with a standard deviation of 0.024, as shown in Figure S4 (See Supl. Mat. S4). As expected from unsteady simulations, the Cd fluctuates around a mean value. ^25, 36^ This fluctuation reflects the model’s ability to capture a broader range of turbulence scales, particularly in the wake region. The DES model achieves this through its spatial filtering mechanism (Δ _LES_), which dynamically resolves detached vortices in both near-wall and far-wake regions. Accurate prediction of these vortex structures significantly improves the precision of the drag coefficient estimation.

### 3.2 Pressure and velocity field

**Figure 4.**
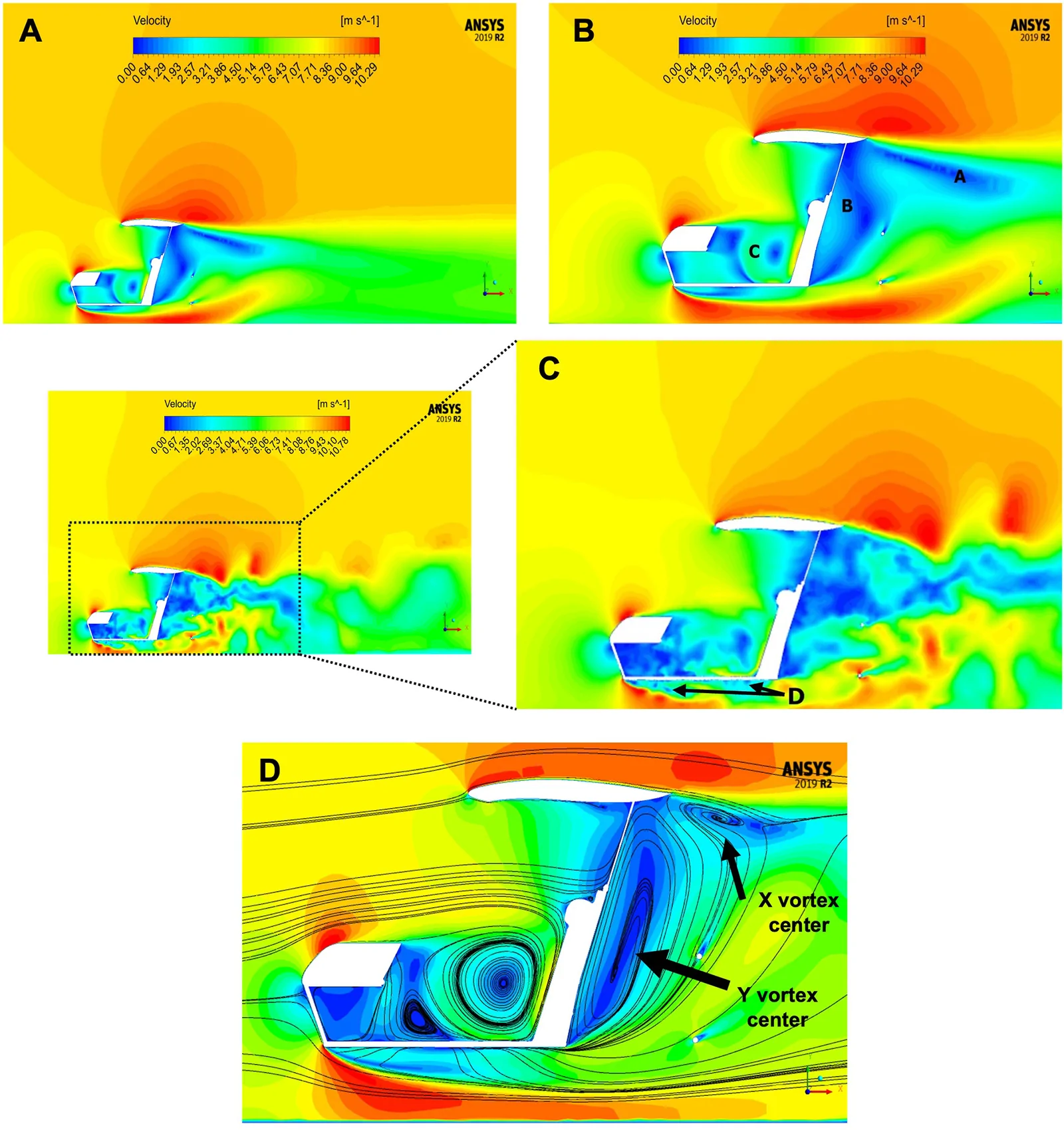
Velocity field and streamlines in symmetry plane. A) Velocity field contour on the symmetry plane using the RANS model; B) Detailed velocity distribution in the near wake region under RANS conditions; C) Velocity field contour on the symmetry plane using the DES model; D) Streamlines on the symmetry plane illustrating flow behaviour around the vehicle with the RANS model.

The aerodynamic behaviour of the Baja SAE vehicle model, as shown in Figure 4, reflects the unique characteristics of a competition-oriented off-road prototype rather than those of a conventional land vehicle. Due to the simplified bodywork and the absence of a front windshield, the flow does not exhibit well-defined aerodynamic features such as coherent recirculation bubbles, separation zones with distinct contours, or consistent vortex formation along roof pillars and between the hood and windshield. Figure 4A presents the velocity field on the symmetry plane using the RANS model. Although flow separation is evident in the near wake, the contours lack definition, revealing two distinct recirculation zones (labelled A and B in Figure 4B). Zone A is elongated in the streamwise direction due to the inertia of flow over the slanted roof, which has an inclination of approximately 5°. ^37^ In contrast, Zone B shows a more compact flow reattachment near the lower rear of the chassis, attributed to the sharp downward change in vehicle geometry. Additionally, the absence of a windshield creates internal recirculation near the driver’s chest and legs (labelled C).

**Figure 5.**
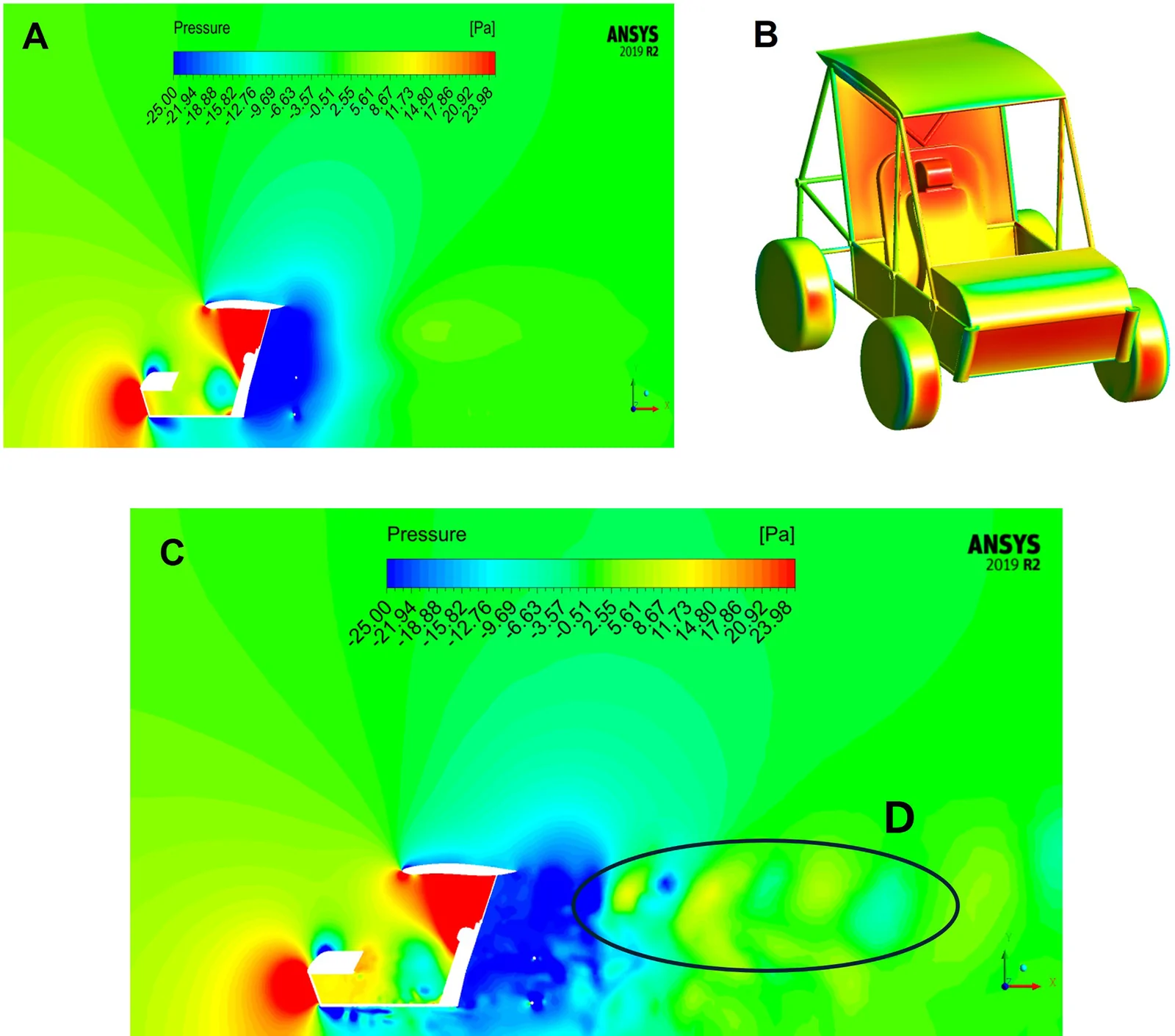
A) Pressure contour in the symmetry plane and on the vehicle surface by the RANS model; B) Pressure distribution on the vehicle surface; C) Pressure contour in the symmetry plane by the DES model.

Figure 5A and 5B show the pressure field in the symmetry plane and on the vehicle’s surface under RANS conditions. Notable pressure gradients are observed beneath the chassis and at key stagnation points—on the nose of the fairing, the front of the wheels, and particularly on the flat canvas behind the driver’s seat. The latter acts as a normal surface to the flow, generating a large stagnation region that significantly contributes to drag.

In contrast, the DES model provides a more detailed and realistic depiction of the flow, particularly in the velocity gradients and wake structure ( Figure 4C). This model captures small low-velocity regions near the lower chassis (Zone D), where the boundary layer develops complex flow behaviour not resolvable by RANS due to its time-averaging limitations. Figure 5C shows the corresponding pressure field obtained by the DES model, where enhanced detail is seen in the near wake. Notably, alternating high- and low-pressure zones are observed near the rear roof region, approximately 1 m downstream (Zone E), reminiscent of the von Kármán vortex street. ^38^

Streamline analysis based on the steady-state RANS model is presented in Figure 4D. Two prominent counter-rotating vortices (labelled X and Y) emerge in the near wake, in agreement with vehicle aerodynamics literature by Hucho ^37^ and Ahmed. ^39^ The upper rear flow conforms to the behaviour of a squareback vehicle with a slant angle α = 5°, as expected. The lower rear wake shows slight deviations due to the chassis’ upward inclination but still produces flow dynamics consistent with squareback-type wake structures, particularly in the formation of the Y-vortex that remains attached to the chassis.

It is important to note that streamline visualisation using the transient DES model is not included, as the temporal discretisation inherent to DES captures rapidly evolving multi-scale turbulence in the wake. These dynamic variations disrupt streamline continuity across time steps, making it impractical to depict a representative streamline field in the near wake using this model.

### 3.3 Vorticity field analysis in the near and far wake

**Figure 6.**
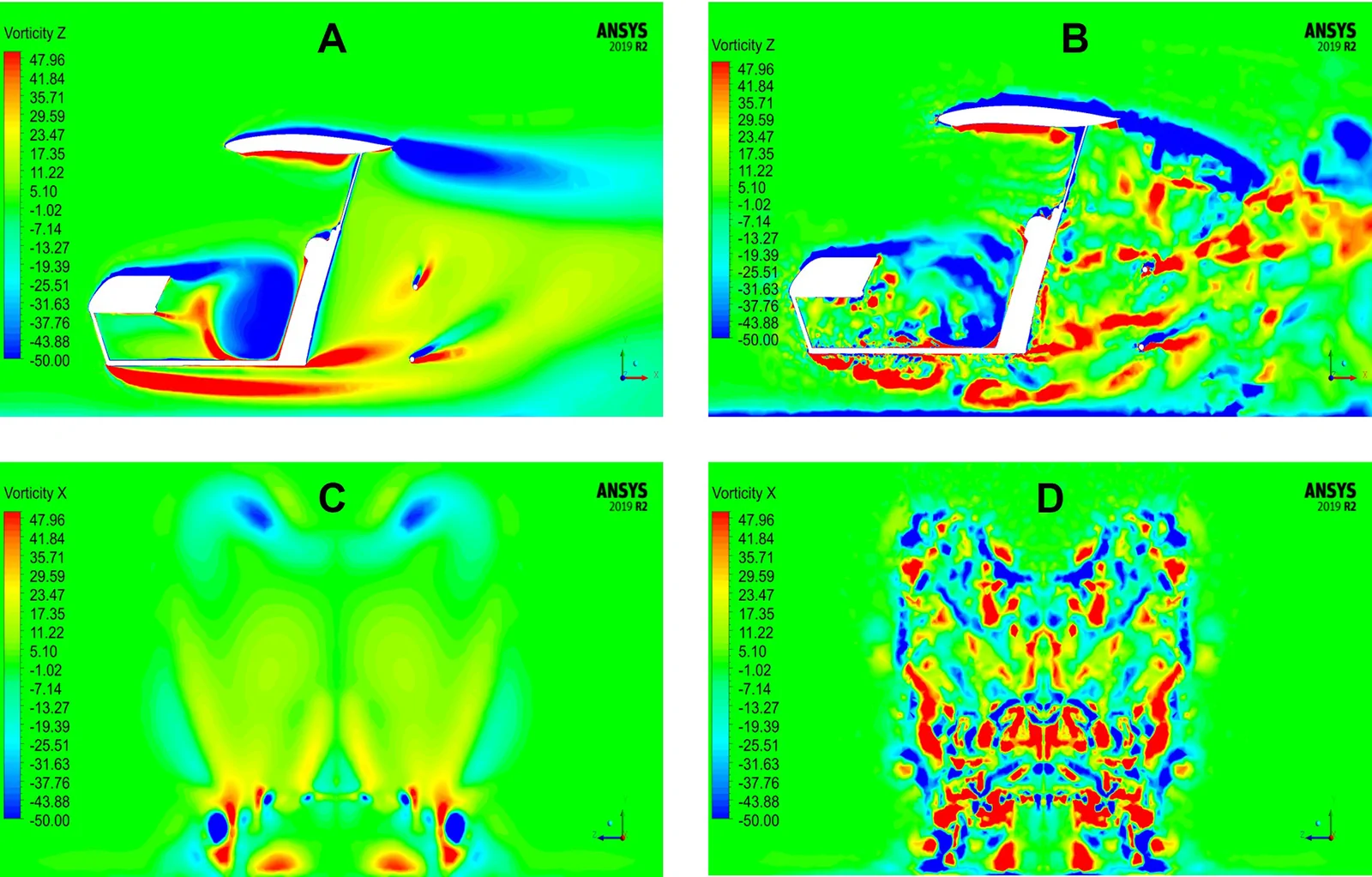
A) Vorticity field contour on the symmetry plane for the RANS model; B) Vorticity field contour on the symmetry plane for the DES model; C) Detailed vorticity field contour in the near wake of the vehicle for the RANS model; D) Detailed vorticity field contour in the near wake of the vehicle for the DES model.

As previously discussed, turbulence models provide a valuable approximation of the flow dynamics around complex geometries, such as a Baja SAE vehicle. Figure 6 presents the longitudinal vorticity contours on the symmetry plane for both the RANS ( Figure 6a) and DES ( Figure 6b) models. In both cases, the near and far wake exhibit similar vortex trajectories and lengths; however, the DES model offers a more detailed representation of vortex structures due to its hybrid formulation. It captures both large-scale and small-scale vortices with greater clarity and spatial resolution.

The RANS model succeeds in identifying the principal vortex structures formed behind the vehicle ( Figure 6C), but it lacks accuracy in resolving their intensity, propagation, and dissipation downstream. This is attributed to the nature of the RANS approach, which models all turbulent scales via statistical averaging, limiting its ability to depict transient or fluctuating phenomena. In contrast, the DES model by blending RANS near walls with LES in detached regions captures a broader spectrum of turbulent scales ( Figure 6D). As a result, the DES simulation reveals a denser and more realistic distribution of vortical structures throughout the wake, particularly in the rotor and downstream zones.

Moreover, the application of the DES model clearly enhances the capture of both micro- and macro-scale vortices along the entire wake region. This improved resolution directly contributes to more accurate predictions of flow field variables, including the vehicle’s drag coefficient and streamline behaviour. The key differences in performance between these turbulence models arise from two fundamental features. First, the RANS model employs a statistical averaging method, decomposing flow variables into mean and fluctuating components, and is applied in a zonal fashion i.e., across the entire computational domain as predefined by the user. Second, the DES model operates as a wall-distance-based method. It dynamically transitions from RANS to LES depending on the distance of the mesh element from the wall and the local grid size, using a spatial filtering mechanism (∆ _LES_) and requiring mesh adaptation to function effectively.

### 3.4 Theoretical framework of vorticity dynamics

Figures 6a and 6b show the vortex structures in the near wake, revealing stretching and diffusion behaviour consistent with classical vorticity dynamics. In the RANS approach, small and medium turbulence scales are entirely modelled, so the analysis is limited to averaged large-scale vorticity structures. According to Bernard and Wallace, ^40^ the steady-state, averaged vorticity transport equation is expressed as:

$$\frac{D{\bar{\omega }}_{i}}{\mathrm{Dt}}={\bar{\omega }}_{j}\frac{\partial {\bar{u}}_{i}}{\partial x_{j}}+\bar{\omega \prime_{j}\frac{\mathrm{\partial u}\prime_{i}}{\partial x_{j}}}+\upsilon \frac{\partial^{2}{\bar{\omega }}_{i}}{\partial x_{j}^{2}}$$

**Equation 2.** Averaged vorticity transport equation.

Here, the left-hand side represents the convection of mean vorticity. The first term on the right-hand side corresponds to stretching due to the mean velocity field, the second term to stretching from turbulent fluctuations, and the third term to viscous diffusion. In RANS models using eddy-viscosity closures (such as Spalart–Allmaras), the fluctuating term is not directly resolved. Instead, the averaged vorticity field is derived from the velocity field obtained via the RANS equations.

This leads to a simplified form of the vorticity transport equation:

$$\frac{D{\bar{\omega }}_{i}}{\mathrm{Dt}}={\bar{\omega }}_{j}\frac{\partial {\bar{u}}_{i}}{\partial x_{j}}+\nabla \times \left(\nabla v_{t}\times \nabla \bar{u}\right)$$

**Equation 3.** Vorticity transport equation.

In this form, the two main physical mechanisms that influence the development of the mean vorticity field are stretching and eddy-viscosity-based $\bar{\omega }$ diffusion. The stretching term governs the rotation and amplification of vortical structures as they evolve downstream, while the diffusion term governs their spatial dispersion. This theoretical framework explains observed phenomena such as reconnection and cancellation of averaged vortical structures in the near wake.

### 3.5 3D Visualization of vortex structures

The generation and transport of vortex structures in the near and far wake of a vehicle can significantly affect its aerodynamic performance. These vortices, resulting from the interaction between the airflow and solid surfaces, are inherently three-dimensional phenomena. Therefore, visualising their evolution and intensity in 3D during post-processing is essential to identify optimisation strategies for vehicle components such as the chassis, fairing, or other elements influencing aerodynamic efficiency.

One widely used method for vortex visualisation is the Q-criterion, which is derived from the second invariant of the velocity gradient tensor, defined as:

$$Q=\frac{1}{2}\left({\left\|\Omega \right\|}^{2}-{\left\|S\right\|}^{2}\right)$$

**Equation 4**. Mathematical definition of the Q-criterion for vortex visualization.

Where S is the symmetric part of the velocity gradient tensor (the rate of strain), and Ω is the antisymmetric part (the vorticity tensor). A region where Q>0 indicates that the local vorticity magnitude dominates over the strain rate, and therefore represents the presence of a vortex. ^41^ Compared to planar vorticity iso-contours, the Q-criterion allows for a more detailed and three-dimensional visualisation of flow dynamics.

**Figure 7.**
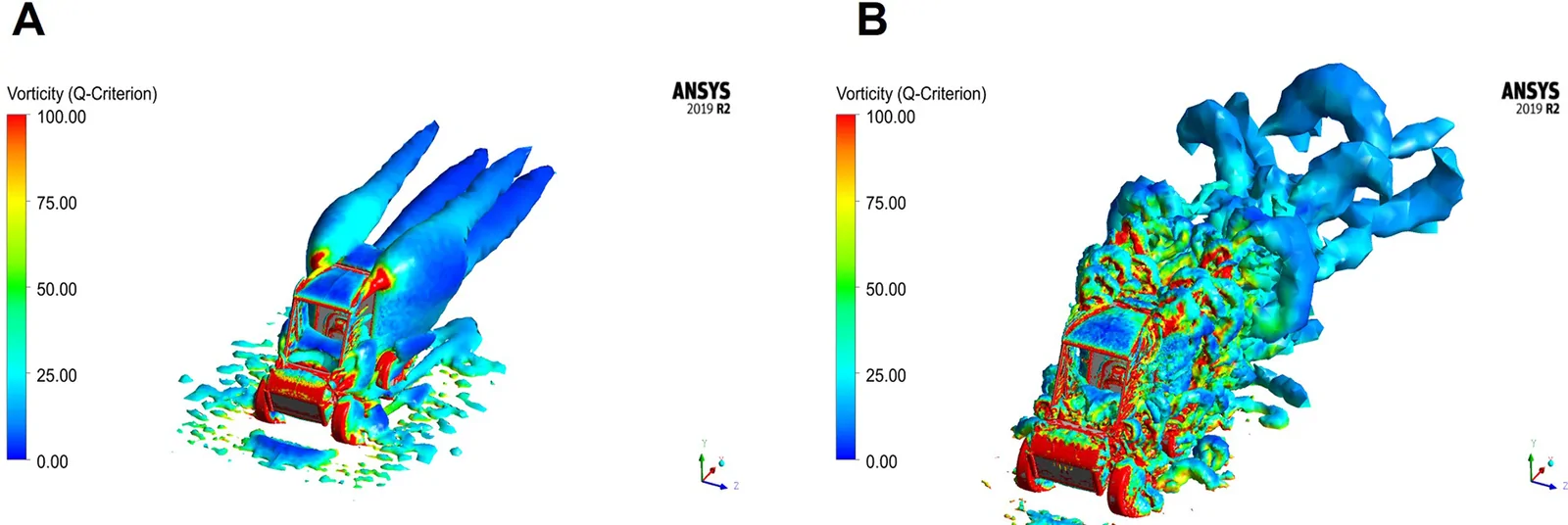
Isocontours of Q-criterion showing vortex structures around the vehicle and in the near wake region. A) RANS model colored by turbulence intensity; B) DES model colored by turbulence intensity.

Figure 7 presents a comparison of vortex structures obtained using the Q-criterion with both RANS (a) and DES (b) turbulence models. In both cases, the main large-scale vortex structures in the near and far wake of the vehicle are consistent with the aerodynamic principles proposed by Hucho. ^37^ These include counter-rotating vortices originating from the vertical uprights that extend from the roof towards the far wake, paired vortices resulting from the interaction between lateral and underbody flows, and small-scale vortices formed around the vehicle’s tires.

The key distinction between the two models lies in their ability to resolve the evolution of these vortex structures. While the RANS model captures the origin of large-scale vortices and propagates them through a spatial averaging process, the DES model introduces both spatial and temporal discretisation. This enables more accurate resolution of both the location and evolution of vortical structures in the near wake, and allows for the capture of smaller, secondary vortices that RANS tends to neglect.

Figure 7 further illustrates these phenomena, showing isocontours of the Q-criterion colored by turbulence intensity. High-intensity vortices are observed at the front fairing, the struts, and the frontal areas of the front wheels regions where vortex formation initiates (RANS model in Figure 7A and DES model in Figure 7B). This behaviour is attributed to specific design characteristics of the Baja SAE vehicle: flat metal panels in the front fairing, uncovered structural struts formed from a single pipe frame integrated into the chassis, and wheels that extend beyond the width of the chassis, which is common in high performance of all-terrain vehicles (ATVs).

## 4. Discussion

The comparative analysis of turbulence models in this study reveals critical insights into the aerodynamic behaviour of the Baja SAE vehicle. The drag coefficient (Cd) computed using the RANS model yielded a value of 1.290 ± 0.005, demonstrating numerical stability through its low standard deviation. However, when using the hybrid RANS-LES (DES) model, the Cd decreased to 1.249 ± 0.024, indicating a more accurate estimation of aerodynamic drag due to the DES model’s capability to resolve a broader range of turbulence scales. This improvement aligns with theoretical expectations for unsteady turbulence modelling ^42^ and validates the utility of DES in external vehicle aerodynamics.

Visualisations of the velocity field and pressure distribution further highlight the enhanced resolution offered by the DES model. The RANS results reveal two distinct recirculation zones in the near wake—Zone A, influenced by the inertia of the upper flow, and Zone B, formed by abrupt geometric changes in the rear lower chassis. However, the DES model resolves finer details of low-velocity regions near the underbody ( Figure 4D), as well as alternating pressure zones at the rear roofline indicative of von Kármán vortex shedding, a phenomenon not captured by RANS. These results confirm that DES enables a richer description of flow structures around the vehicle, especially in wake regions critical for drag generation.

Additionally, streamline analysis of the RANS solution identified two counter-rotating vortices (X and Y) in the near wake ( Figure 4D), consistent with known behaviour in squareback vehicle geometries. ^37, 39^ The Baja SAE vehicle exhibits a slant angle of ~5°, resembling Ahmed-type body configurations. Despite geometric differences in the underbody, a similar vortex formation pattern is observed, although the recirculation region remains more closely attached to the chassis.

Overall, the results demonstrate that while the RANS model provides a reasonable first-order approximation of aerodynamic characteristics, the DES model offers superior fidelity in capturing transient vortex structures, wake turbulence, and drag prediction. These findings are essential for future design improvements aimed at optimising the aerodynamic performance of non-commercial, competition grade vehicles like the Baja SAE.

## 5. Conclusion

This study presents a comparative aerodynamic analysis of a Baja SAE vehicle using RANS and hybrid RANS-LES (DES) turbulence models. While RANS provided stable and computationally efficient results, it underestimated vortex dynamics and turbulent fluctuations in the wake. DES, by contrast, captured a richer spectrum of vortex structures particularly in the near and far wake resulting in a more accurate prediction of aerodynamic forces. DES predicted a lower drag coefficient (1.249 ± 0.024) than RANS (1.290 ± 0.005) and delivered higher spatial detail in velocity, pressure, and vortex fields. Q-criterion and streamline visualizations confirmed its ability to resolve a broader range of turbulent scales, offering deeper insights for aerodynamic optimization. Future work will focus on coupling DES with shape optimization techniques and wind tunnel validation to further enhance the aerodynamic performance of competitive off-road vehicles.

## Use of artificial intelligence

We acknowledge that artificial intelligence tools (ChatGPT, OpenAI) were employed exclusively to assist with the revision of the English language and to improve the clarity of the writing. All scientific content, analyses, and conclusions are entirely the work of the authors.

## Data Availability

The materials supporting the findings of this study are available in the Zenodo repository: https://doi.org/10.5281/zenodo.17295975. ^43^ These materials include the ANSYS simulation files used to generate the computational results presented in the paper (geometry, mesh, boundary conditions, and solver configuration). No external datasets were used in this study, as all results derive from numerical simulations developed by the authors. The files are shared under a Creative Commons Attribution 4.0 International (CC BY 4.0) license, allowing reuse and adaptation with proper citation. Due to file size limitations, the complete raw simulation output can be provided upon reasonable request to the corresponding author. Additional materials supporting this study are available in the Zenodo repository: DOI: https://doi.org/10.5281/zenodo.17295975. ^43^ This record includes the ANSYS project files used in the numerical simulations described in the manuscript, comprising the 3D geometry model of the vehicle, computational mesh file, boundary condition setup, solver configuration, and post-processing parameters. All files are shared under a Creative Commons Attribution 4.0 International (CC BY 4.0) license.
