# Aerodynamic Performance of a Baja SAE Vehicle Using Hybrid RANS-LES Approach

**DOI:** 10.12688/f1000research.171076.3

**Published:** 2026-07-25

**Authors:** Sergio A. Ardila Gomez, Jessica G. Maradey Lazaro, Jhon J. Quiñones, Deisy Y. Rodriguez Sarmiento

**Affiliations:** 1 Universidad Autonoma de Bucaramanga, Bucaramanga, Santander, Colombia; 2 Purdue University, West Lafayette, Indiana, USA

**Keywords:** Baja SAE vehicle aerodynamics, Hybrid RANS LES turbulence modeling, Computational Fluid Dynamics CFD, Drag and lift coefficients, Flow separation and wake dynamics

## Abstract

This study presents a high-fidelity computational investigation of the aerodynamic performance of a Baja SAE off-road vehicle using a hybrid Reynolds-Averaged Navier–Stokes (RANS) and Large Eddy Simulation (LES) turbulence modeling approach. The methodology combines the Spalart–Allmaras RANS model for near-wall flow treatment with Detached Eddy Simulation (DES) for resolving large-scale unsteady turbulent structures in the vehicle’s wake. A detailed computational domain and refined meshing strategy were implemented using ANSYS Fluent, including mesh adaptation based on the LES filter size (Δ = 23.5 mm) and mesh independence validation.

Simulations were performed under steady (RANS) and unsteady (DES) conditions at 30 km/h, yielding a drag coefficient (Cd) of 1.290 for RANS and 1.249 for DES. While RANS provided stable results with low variance (σ = 0.005), the DES model captured transient phenomena such as vortex shedding and near-wake recirculation with higher accuracy (σ = 0.024), enhancing the prediction of flow separation zones and aerodynamic forces. In this work, the analysis focuses on the drag coefficient as a primary indicator of wake-induced aerodynamic losses, rather than providing a complete aerodynamic force and moment characterization. Pressure and velocity field analyses revealed improved resolution of stagnation zones and vortex dynamics under the DES framework, particularly around the roof, rear, and underbody regions.

The Q-criterion visualization showed that DES allowed the identification of both large-scale and fine-scale vortex structures in the near and far wake, offering a comprehensive representation of turbulence intensity and flow instabilities. These findings confirm the suitability of hybrid RANS–LES methods for aerodynamic optimization of complex vehicle geometries, providing enhanced predictive capabilities compared to traditional steady-state models.

## 1. Introduction

The Baja SAE competition is an international engineering challenge that promotes the design, analysis, and manufacturing of off-road vehicles by university students under real-world constraints. Participating teams must address key aspects such as safety, reliability, regulatory compliance, cost-efficiency, and overall performance. ^1^ Beyond technical development, the project fosters management and teamwork skills, aligning with educational objectives outlined by the Accreditation Board for Engineering and Technology (ABET). ^2, 3^

The vehicle must meet critical requirements including robustness, ease of maintenance, driver accessibility, and the ability to traverse rough terrain—all within a limited budget of $2,500. ^4^ To achieve optimal performance, technical efforts focus on chassis design, material selection, and the improvement of mechanical systems such as suspension and steering. ^1^ Structural integrity is assessed using Finite Element Analysis (FEA), which enables virtual evaluation of stress, deformation, and vibration modes under various loading scenarios typical in Baja SAE events, such as acceleration, endurance, and impact tests. ^4, 5^ Static and dynamic analyses help prevent structural failure and enhance occupant safety, while software tools like ANSYS, COMSOL, or LS-DYNA are used for advanced simulations including frontal crashworthiness and torsional stiffness validation. ^6, 7^

In addition to structural strength, aerodynamic performance plays a key role in reducing drag, improving stability, and optimizing fuel efficiency. Aerodynamic assessments can be conducted experimentally or numerically, with Computational Fluid Dynamics (CFD) emerging as a cost-effective alternative to wind tunnel or on-road testing. ^8, 9^ CFD allows accurate prediction of flow behaviour including drag, lift, vortex shedding, and pressure distributions using only the vehicle’s 3D geometry. This methodology accelerates the design process and reveals flow inefficiencies that may be overlooked through conventional design approaches. ^10, 11^ Drag force is a major contributor to fuel consumption at high speeds, ^8^ while lift affects tire adherence and vehicle stability. Understanding these parameters is essential for performance optimisation. Table 1 summarise recent studies involving FEA and CFD in the design of Baja SAE vehicles.

**Table 1.** Overview of relevant studies involving Finite Element Analysis (FEA) and Computational Fluid Dynamics (CFD) applied to structural and aerodynamic optimisation of Baja SAE vehicles.

Reference & year	Methods used	Key findings
Zhang et al., 2024 ^12^	Vehicle Dynamics Modeling, sensor instrumentation	Validated vehicle dynamics, improving prototype design
Franco-Camacho et al., 2020 ^13^	Multi-body dynamics, FEA for suspension & chassis	Optimized suspension and chassis, validated handling & strength
Zainal et al, 2018 ^14^	FEA structural & modal analysis with experimental validation	Optimized resonance modes, improving structural safety
Bhardwaj et al., 2023 ^15^	FEA design & optimization of steering assembly	47% weight reduction, enhanced handling
Vinod et al, 2017 ^16^	Modal Analysis of chassis with CAD and ANSYS	Identified natural frequencies, improved durability
Khan et al., 2023 ^17^	CAD and power transmission simulation for driveshaft	Optimized driveshaft design for off-road 4x4 system
Kumar et al., 2024 ^18^	CAD and FEA design of customized brake caliper	Created brake caliper supporting 315,645 Nm torque
Maradey et al., 2019 ^1^	CFD Simulations (ANSYS Fluent) for aerodynamics analysis	Improved airflow understanding, drag, lift, pressure distribution
Silva et al., 2023 ^19^	Suspension design and MSC Adams Car© (with aerodynamic considerations)	Double-stage springs improved dynamic response and aerodynamics
Trinadh Raju et al.,2025 ^20^	CFD (ANSYS) for aerodynamics; FEA for impact analysis	Drag force 185N at 60km/h; validated structural integrity
Abu Farjad & Predators Racing, 2025 ^21^	CFD (Ansys Fluent) and FEA for drag reduction & optimization	5% drag reduction and 6.5kg weight saving through simulation

CAD (Computer Aided Design).

Computational Fluid Dynamics (CFD) has become a vital resource in the aerodynamic development of Baja SAE vehicles, enabling accurate analysis of complex flow phenomena—such as drag, lift, vortex shedding, and pressure distribution—using only the vehicle’s 3D geometry. By eliminating the need for physical prototypes, CFD accelerates design iterations, reduces costs, and enhances precision in flow prediction. It supports the optimisation of key components like bodywork, intakes, and cooling systems, improving thermal performance, reducing aerodynamic drag, and increasing overall vehicle stability. Additionally, CFD visualisations of pressure and velocity fields provide critical insights during early development, making it a cost-effective and powerful alternative to experimental methods like wind tunnel or road testing ^1, 22– 24^ The general CFD workflow followed in this study is summarised in Figure S1 (see Supplementary Material 1).

To simulate these flow phenomena with sufficient accuracy and computational efficiency, particularly in regions with flow separation and vortex dynamics, the selection of an appropriate turbulence model is essential. Turbulent flows pose significant challenges due to their inherent unsteadiness and wide range of scales. While Direct Numerical Simulation (DNS) offers the most accurate solution, it is computationally prohibitive for full-vehicle analysis. Therefore, turbulence modeling strategies such as Reynolds-Averaged Navier–Stokes (RANS), Large Eddy Simulation (LES), and hybrid RANS–LES frameworks are widely used. RANS is commonly applied near walls for its efficiency, while LES resolves large-scale structures in regions such as wakes and shear layers. ^25, 26^

Hybrid models combine both approaches, leveraging the strengths of RANS in boundary layers and LES in separated flow regions to balance accuracy and computational cost. A critical component of these models is the transition mechanism between RANS and LES zones, managed via zonal interfaces or dynamic blending functions based on local flow properties. ^25^ Among the most commonly used hybrid methods are Detached Eddy Simulation (DES), Delayed Detached Eddy Simulation (DDES), and Scale-Adaptive Simulation (SAS). DDES improves stability by preventing premature switching and reducing sensitivity to grid resolution, ^27^ while SAS dynamically adjusts turbulence length scales based on local conditions, eliminating the need for manual interface definitions. ^26^ A comparative overview of these turbulence models is provided in Figure S2 (see Supplementary Material 2).

While a complete aerodynamic characterization of ground vehicles typically involves multiple force components (drag, lift, and side force) and aerodynamic moments, the present study focuses on drag as a representative parameter of wake-induced losses and turbulence model performance. This approach is consistent with the objective of evaluating the capability of hybrid RANS–LES methods to resolve flow separation and vortex dynamics, which are the dominant contributors to aerodynamic drag in bluff-body configurations such as Baja SAE vehicles.

This article outlines the aerodynamic analysis methodology for a Baja SAE vehicle. Section 2 describes the geometry, meshing strategy, RANS and DES turbulence models, and simulation setup. Section 3 presents results comparing both models in terms of drag prediction, flow fields, vorticity, and 3D vortex structures. Despite the growing use of CFD in Baja SAE vehicle development, limited attention has been given to the comparative performance of turbulence modeling approaches in capturing wake dynamics. This study addresses this gap by evaluating the capability of hybrid RANS–LES methods relative to steady RANS simulations.

## 2. Methods

This section first describes the computational setup and then presents the rationale behind the selected turbulence modeling approach.

### 2.1 Geometry and computational domain

**Figure 1.**
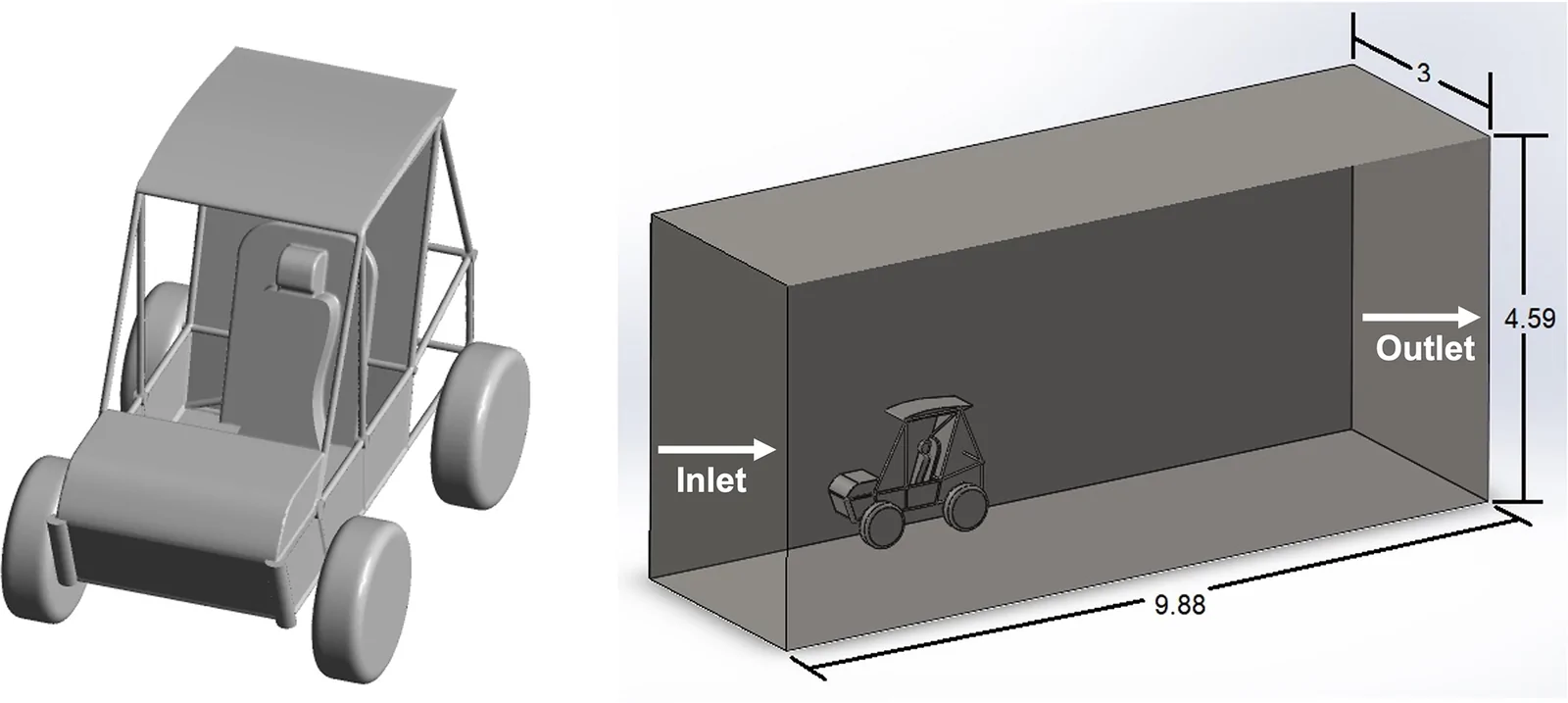
Vehicle geometry and computational domain.

The 3D model of the Baja SAE vehicle used in this study is shown in Figure 1A. It was developed based on the geometric specifications listed in Table 2. To reduce computational cost, several geometric details—particularly in the engine bay, underfloor region, and driver compartment—were simplified. Additionally, aerodynamic refinements were implemented on the vehicle’s front and roof areas to improve flow behaviour and streamline continuity.

**Table 2.** Vehicle and computational domain dimensions.

Baja SAE		Computational domain	
Dimension	Value	Dimension	Value
**Height**	1530 mm	Model incident flow	1.3 x vehicle length
**Width without wheels**	856.8 mm	Model – outflow	3 x vehicle length
**Total length**	1883.71 mm	Total width	7 x vehicle width
**Front area (half )**	0.60033 m ^2^	Model – roof	3 x vehicle height
**Characteristic length (between axis)**	1175.85 mm		

The computational domain dimensions are shown in Figure 1B and summarized in Table 2, and were selected following established guidelines from previous aerodynamic simulations to avoid influence from far-field recirculation and ensure numerical accuracy. ^28, 29^ A symmetry boundary condition was applied along the lateral plane, which effectively halves the simulation domain when the flow is symmetric, significantly reducing computation time without compromising fidelity. ^30^

A) 3D CAD model of the Baja SAE vehicle; B) Schematic of the computational domain with dimensions in meters.

### 2.2 Meshing

**Figure 2.**
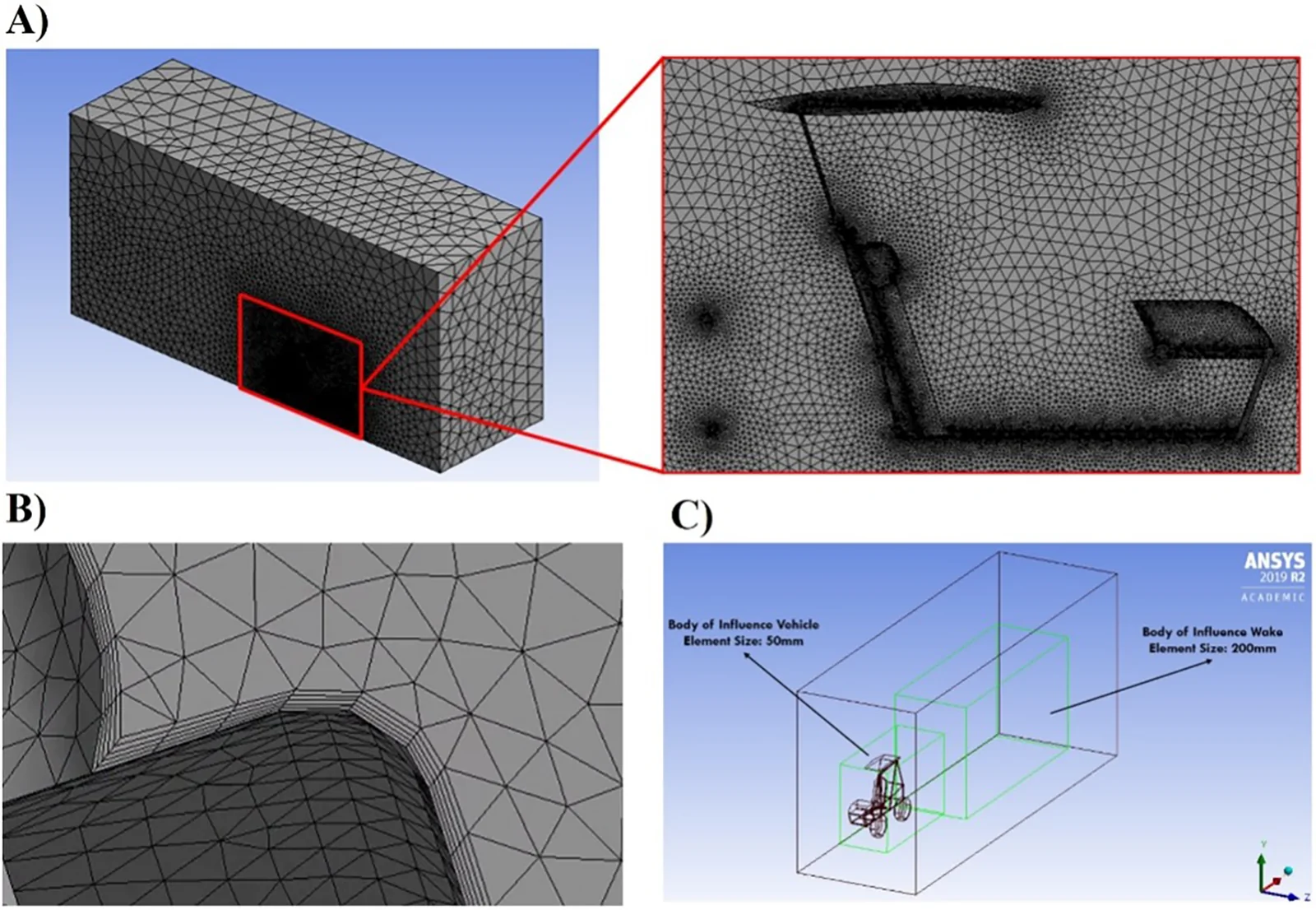
A) Computational domain and vehicle mesh; B) Detail of the prism layer on the vehicle; C) Body of influence boxes.

Figure 2A shows the hybrid mesh generated in the ANSYS meshing module. This mesh consists of tetrahedral elements throughout the domain and prism layers near the vehicle surface, where the turbulent boundary layer is expected ( Figure 2B). The prism layer was configured with an initial height of 0.393 mm, a growth rate of 1.12, and five total layers, resulting in a non-dimensional wall distance (Y ^+^) below 5. Prism elements were also applied to the tire surfaces.

To improve resolution around the vehicle and its wake, two box-shaped bodies of influence were added ( Figure 2C), using element sizes of 50 mm near the vehicle and 200 mm in the wake region. These refinements help capture vortical structures at multiple scales and ensure a smooth transition from near-field to far-field mesh densities.

A mesh independence study was conducted using six mesh configurations with total cell counts ranging from 800,000 to 10 million. The drag coefficient (Cd) was used as the convergence criterion, and mesh independence was achieved with approximately 1.16 × 10 ^6^ elements. Mesh quality was evaluated using the Jacobian determinant, with most elements scoring between 0.7 and 0.95, which is acceptable for accurately resolving boundary layer phenomena. Results of the mesh independence analysis are presented in Figure S3 (see Supplementary Material 3).

### 2.3 RANS Model implementation

In the implemented model, the airflow was assumed to be incompressible (isothermal), Newtonian, and three-dimensional. The working fluid was dry air under standard conditions (T = 25°C, 1 atm), corresponding to a Reynolds number of 6.7 × 10 ^5^, based on the vehicle’s wheelbase.

The boundary conditions were defined as follows: a uniform inlet velocity of 30 km/h (8.33 m/s) was imposed at the domain entrance ( Figure 1B), while the outlet was set to constant atmospheric pressure. The lateral, upper, and symmetry planes were treated as symmetry boundaries (zero-gradient) to avoid artificially enclosing the wake region and to ensure realistic flow development. The ground, vehicle chassis, and tyres were modelled as stationary no-slip walls.

Simulations were performed using ANSYS FLUENT v19.2 ^31^ under steady-state conditions. The one-equation Spalart–Allmaras turbulence model ^32^ was employed for the RANS formulation due to its efficiency in external aerodynamic flows at high Reynolds numbers and its relatively low computational cost. The transport equation for the model is expressed as Eq 1:

$$\frac{\partial }{\mathrm{\partial t}}\left(\rho \tilde{v}\right)+\frac{\partial }{\partial x_{i}}\left(\rho \tilde{v}u_{i}\right)=G+\frac{1}{\sigma }\left\{\frac{\partial }{\partial x_{j}}\left[\left(\rho \tilde{v}+\mu \right)\;\frac{\partial \tilde{v}}{\partial x_{j}}\right]+C_{b}{\left(\frac{\partial \tilde{v}}{\partial x_{j}}\right)}^{2}\right\}-Y$$

**Equation 1.** Transport equation for the modified turbulent kinematic viscosity ν in the Spalart–Allmaras one-equation turbulence model. ^32^

where *G* represents the production of turbulent viscosity, *Y* is its destruction near the wall due to viscous damping and wall-blocking effects, *σ* and *cb* are empirical constants, and *ν* is the molecular viscosity. A second-order spatial discretisation scheme was used, and pressure–velocity coupling was achieved through the COUPLED algorithm. Convergence was considered achieved when the residuals dropped below 1 × 10 ^−5^ within 5000 iterations.

### 2.4 DES Model adaptation

The hybrid RANS-LES turbulence model DES was implemented in this study to obtain more accurate results and improve the visualization of the vorticity iso-surfaces in the near wake of the Baja SAE vehicle. In the DES model, the flow field can be divided into three regions: Euler Region, RANS Region and LES Region. The boundary layer regions are modelled with unsteady RANS (URANS) and the outer detached vortices are captured with LES. ^33– 35^ The LES region and the called grey zones were calculated based on the guidelines reported on. ^35^

The estimation of the LES filter size (ΔLES) was used as a practical guideline to define mesh refinement regions in the wake. This estimation was based on characteristic geometric and flow scales, particularly the vehicle length, and should be interpreted as an approximate criterion rather than a strict determination of turbulence length scales. The estimation of the LES filter size (ΔLES) was used as a practical guideline to define mesh refinement regions in the wake. This estimation was based on characteristic geometric and flow scales and should be interpreted as an approximate criterion rather than a strict determination of turbulence length scales.

In accordance with standard DES/DDES formulations, the filter size Δ is defined based on the local grid resolution, typically as a characteristic cell dimension (e.g., Δ ≈ V1/3, where V is the cell volume), which is equivalent to the representative grid spacing used to govern the transition between RANS and LES regions.

**Figure 3.**
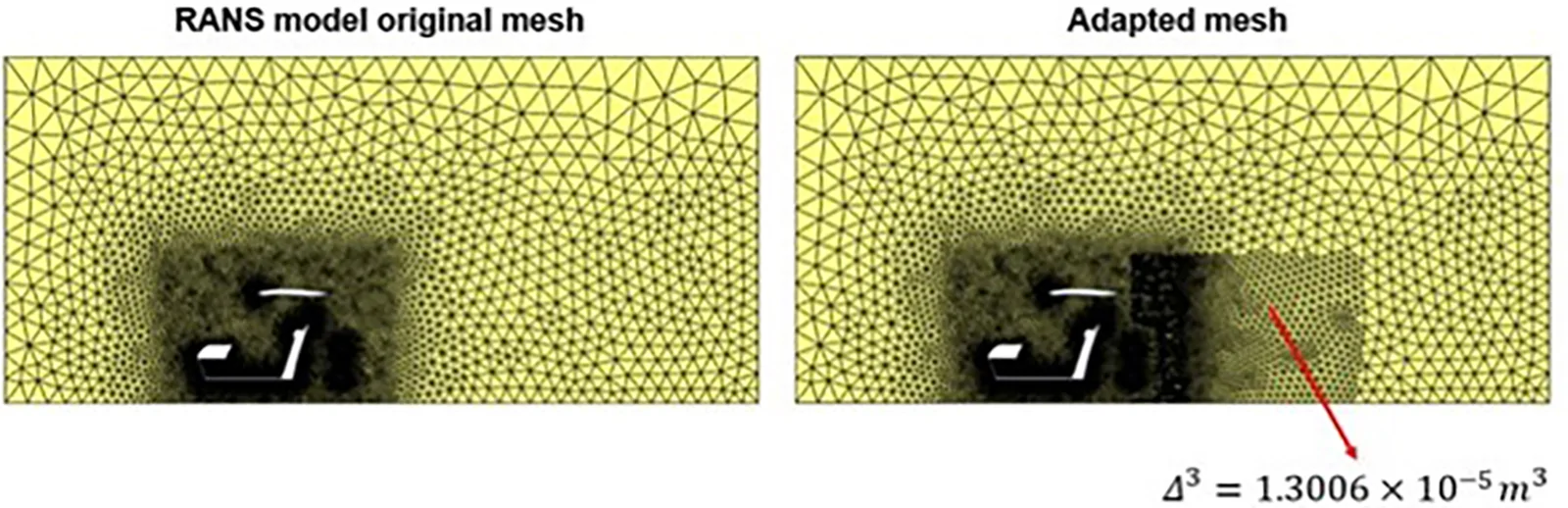
Mesh adaptation from RANS to DES.

In this study, an initial estimate of Δ was obtained using a characteristic length scale of the vehicle to guide mesh design, resulting in Δ ≈ 23.5 mm. This value was used to define refinement regions in areas where flow separation and vortex formation are expected, ensuring adequate spatial resolution of the wake while maintaining computational feasibility within the DES framework. Based on this criterion, the corresponding cell refinement volume (Δ ^3^ ≈ 1.30 × 10 ^−5^ m ^3^) was defined and used to adapt the mesh in ANSYS Fluent ( Figure 3), resulting in a final mesh of approximately 7 million elements.

The time step for the DES simulations is calculated according to ^9^ as ∆t = ∆/Umax = ∆/U∞ = 2.822 × 10 ^−3^ s, where Umax, is the maximum velocity registered in the simulations, which in this case is the velocity of the incident fluid flow. The total time of analysis was determined as t = 4 Lcar/U∞ = 0.904s and 40 iterations per time step were used, where Lcar is the total length of car model.

Although the Reynolds number of the present study falls within a range where steady RANS approaches are often sufficient for predicting global aerodynamic quantities, the use of DES is motivated by the need to better resolve unsteady flow structures in the wake region. Bluff-body flows, even at moderate Reynolds numbers, exhibit complex flow separation and vortex dynamics that benefit from hybrid turbulence modeling approaches, particularly when the objective is to analyses wake behavior in detail.

## 3. Results

### 3.1 Drag coefficient

For the RANS model, the drag coefficient (Cd) was calculated by averaging the values from the final 100 iterations of the steady-state simulation. The resulting Cd was approximately 1.290, with a standard deviation of 0.005, indicating good convergence stability of the RANS solution. In contrast, the hybrid RANS–LES model was implemented under unsteady-state conditions. The time-averaged Cd, computed from the instantaneous values at each timestep, was 1.249, with a standard deviation of 0.024, as shown in Figure S4 (see Supplementary Material S4). As expected in unsteady simulations, the drag coefficient fluctuates around a mean value ^25, 37^ reflecting the transient nature of the flow. These fluctuations are associated with the resolution of time-dependent turbulent structures, particularly in the wake region, rather than representing a direct measure of predictive accuracy. The DES model captures a broader range of turbulence scales through its spatial filtering mechanism (ΔLES), allowing for a more detailed representation of vortex shedding and flow separation phenomena. Consequently, the comparison between RANS and DES results should be interpreted in terms of their relative capability to resolve flow structures and wake dynamics, rather than as an absolute measure of accuracy in drag prediction.

### 3.2 Pressure and velocity field

**Figure 4.**
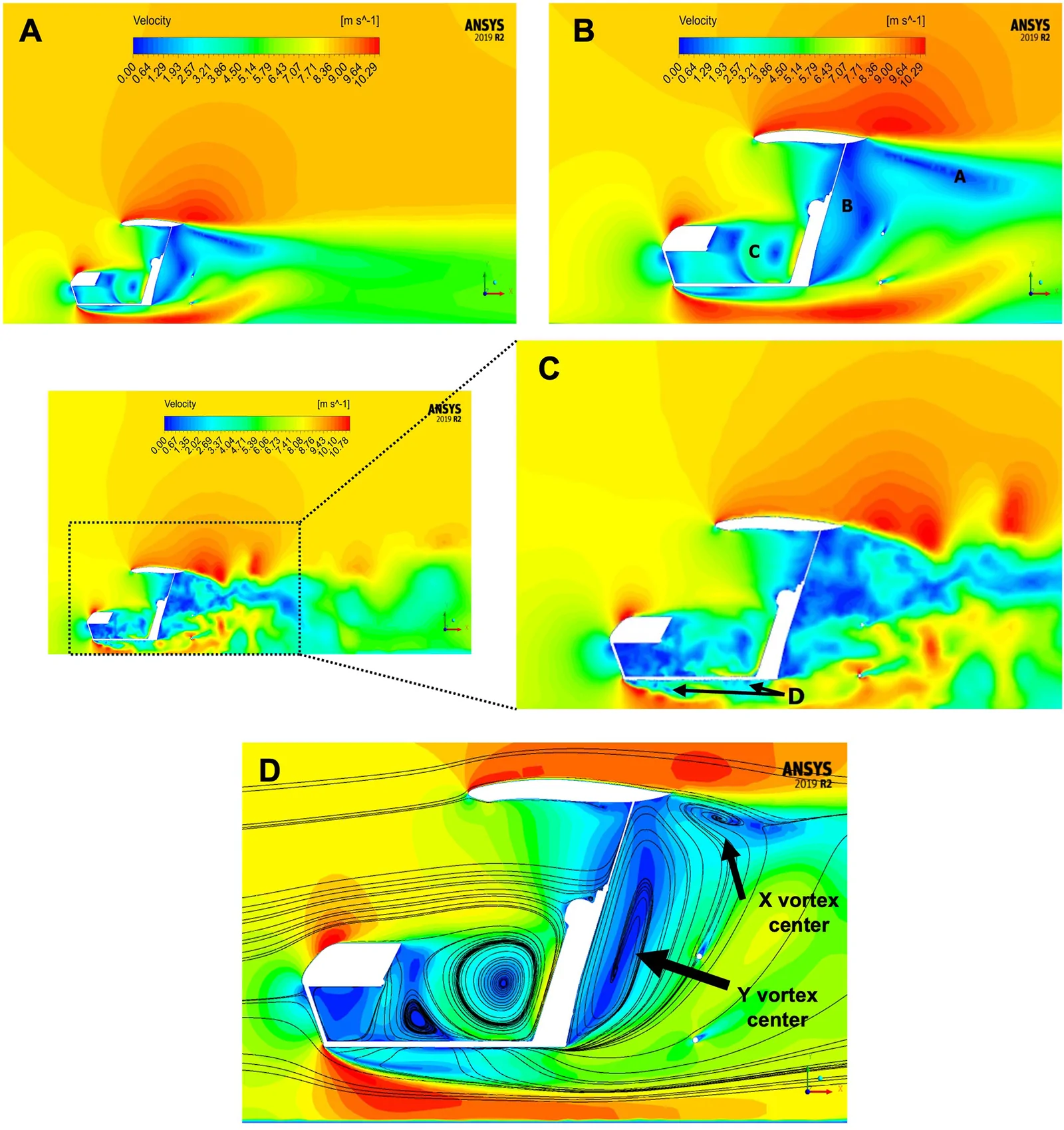
Velocity field and streamlines in symmetry plane. A) Velocity field contour on the symmetry plane using the RANS model; B) Detailed velocity distribution in the near wake region under RANS conditions; C) Velocity field contour on the symmetry plane using the DES model; D) Streamlines on the symmetry plane illustrating flow behaviour around the vehicle with the RANS model.

The aerodynamic behaviour of the Baja SAE vehicle model, as shown in Figure 4, reflects the unique characteristics of a competition-oriented off-road prototype rather than those of a conventional land vehicle. Due to the simplified bodywork and the absence of a front windshield, the flow does not exhibit well-defined aerodynamic features such as coherent recirculation bubbles, separation zones with distinct contours, or consistent vortex formation along roof pillars and between the hood and windshield. Figure 4A presents the velocity field on the symmetry plane using the RANS model. Although flow separation is evident in the near wake, the contours lack definition, revealing two distinct recirculation zones (labelled A and B in Figure 4B). Zone A is elongated in the streamwise direction due to the inertia of flow over the slanted roof, which has an inclination of approximately 5°. ^37^ In contrast, Zone B shows a more compact flow reattachment near the lower rear of the chassis, attributed to the sharp downward change in vehicle geometry. Additionally, the absence of a windshield creates internal recirculation near the driver’s chest and legs (labelled C).

**Figure 5.**
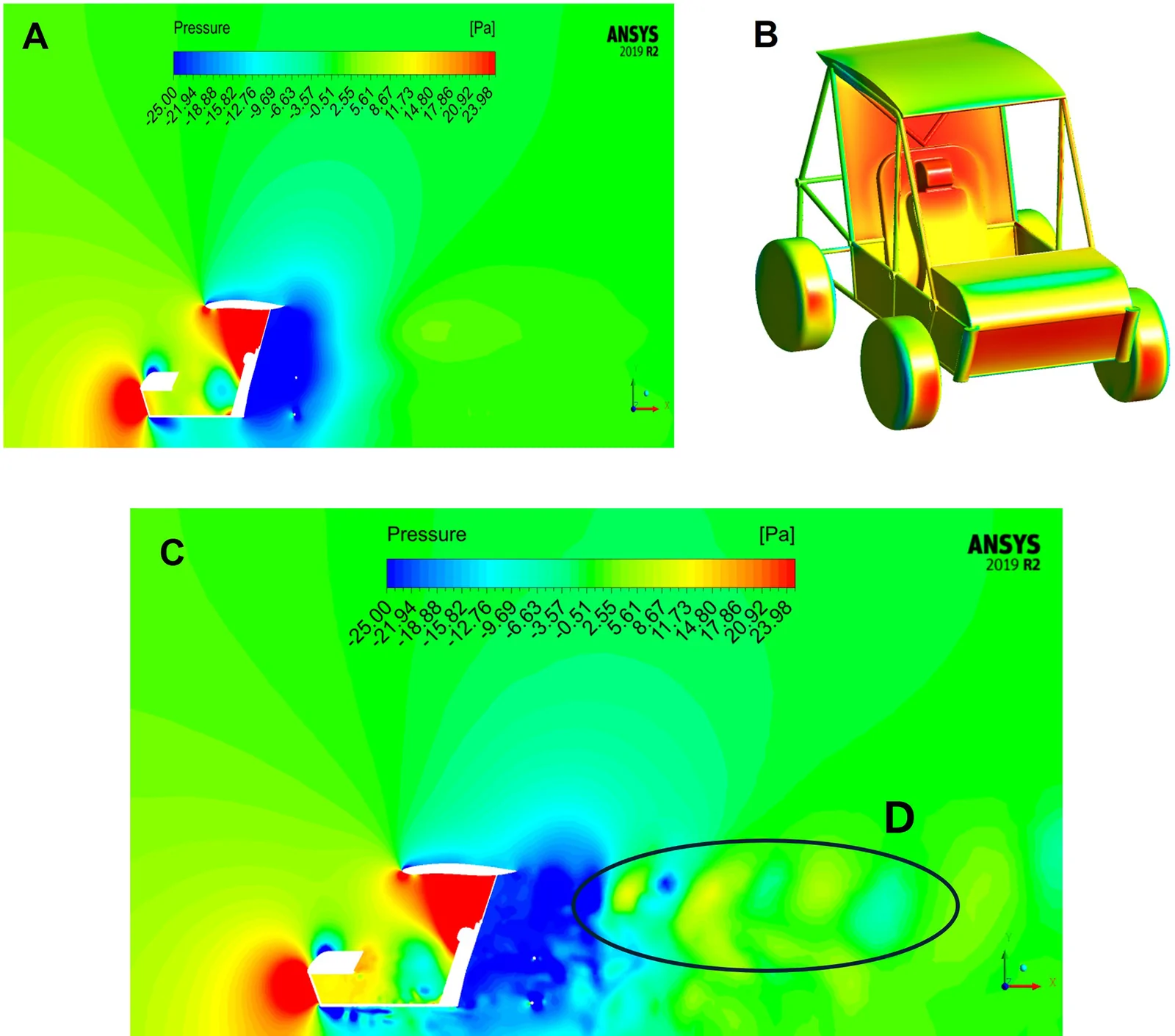
A) Pressure contour in the symmetry plane and on the vehicle surface by the RANS model; B) Pressure distribution on the vehicle surface; C) Pressure contour in the symmetry plane by the DES model.

Figure 5A and 5B show the pressure field in the symmetry plane and on the vehicle’s surface under RANS conditions. Notable pressure gradients are observed beneath the chassis and at key stagnation points—on the nose of the fairing, the front of the wheels, and particularly on the flat canvas behind the driver’s seat. The latter acts as a normal surface to the flow, generating a large stagnation region that significantly contributes to drag.

In contrast, the DES model provides a more detailed and realistic depiction of the flow, particularly in the velocity gradients and wake structure ( Figure 4C). This model captures small low-velocity regions near the lower chassis (Zone D), where the boundary layer develops complex flow behaviour not resolvable by RANS due to its time-averaging limitations. Figure 5C shows the corresponding pressure field obtained by the DES model, where enhanced detail is seen in the near wake. Notably, alternating high- and low-pressure zones are observed near the rear roof region, approximately 1 m downstream (Zone D), suggesting alternating vortex structures in the wake. ^38^

Streamline analysis based on the steady-state RANS model is presented in Figure 4D. Two prominent counter-rotating vortices (labelled X and Y) emerge in the near wake, in agreement with vehicle aerodynamics literature by Hucho ^37^ and Ahmed. ^39^ The upper rear flow conforms to the behaviour of a squareback vehicle with a slant angle α = 5°, as expected. The lower rear wake shows slight deviations due to the chassis’ upward inclination but still produces flow dynamics consistent with squareback-type wake structures, particularly in the formation of the Y-vortex that remains attached to the chassis.

It is important to note that streamline visualisation using the transient DES model is not included, as the temporal discretisation inherent to DES captures rapidly evolving multi-scale turbulence in the wake. These dynamic variations disrupt streamline continuity across time steps, making it impractical to depict a representative streamline field in the near wake using this model.

### 3.3 Vorticity field analysis in the near and far wake

**Figure 6.**
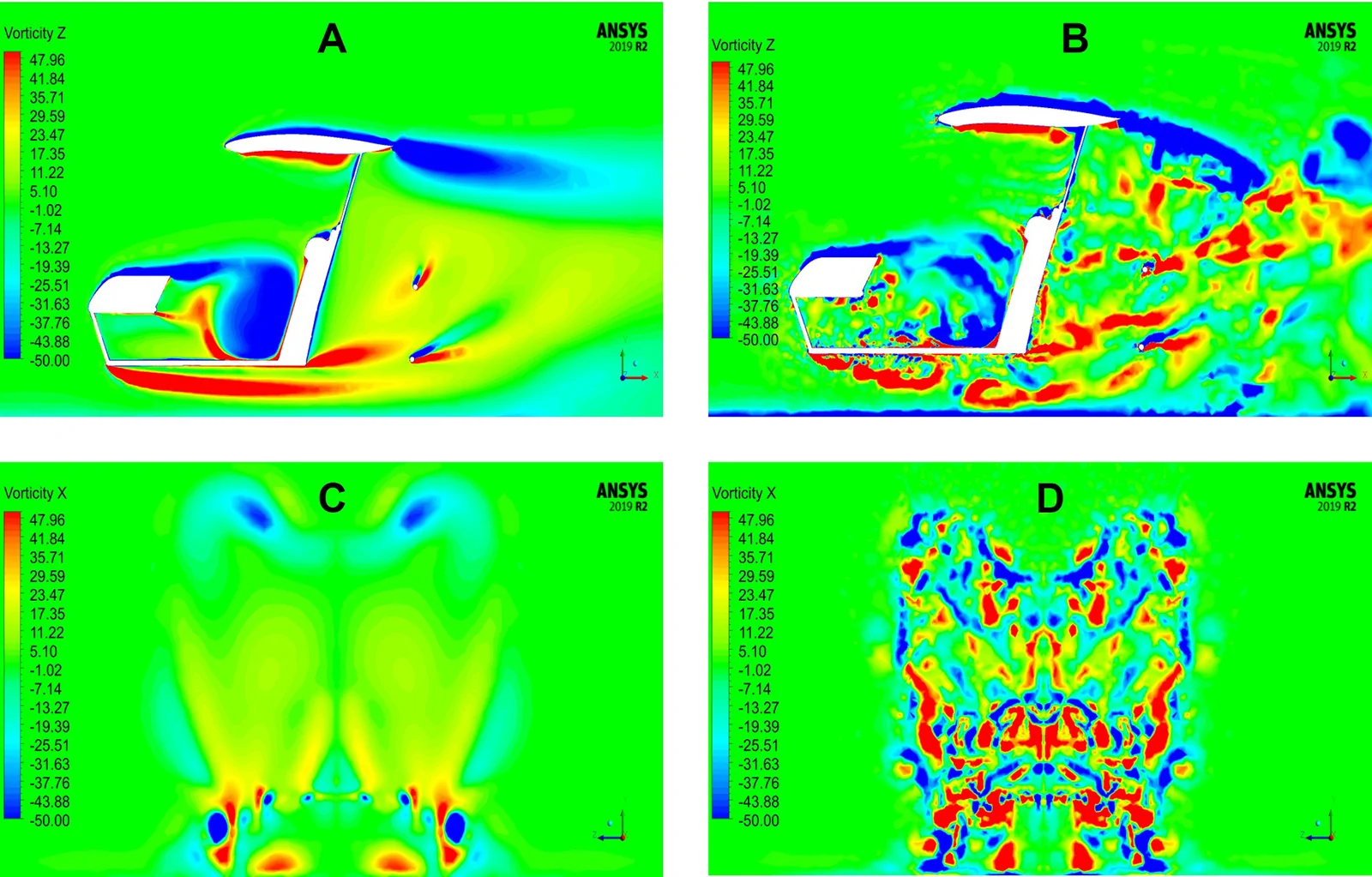
A) Vorticity field contour on the symmetry plane for the RANS model; B) Vorticity field contour on the symmetry plane for the DES model; C) Detailed vorticity field contour in the near wake of the vehicle for the RANS model; D) Detailed vorticity field contour in the near wake of the vehicle for the DES model.

As previously discussed, turbulence models provide a valuable approximation of the flow dynamics around complex geometries, such as a Baja SAE vehicle. Figure 6 presents the longitudinal vorticity contours on the symmetry plane for both the RANS ( Figure 6A) and DES ( Figure 6B) models. In both cases, the near and far wake exhibit similar vortex trajectories and lengths; however, the DES model offers a more detailed representation of vortex structures due to its hybrid formulation. It captures both large-scale and small-scale vortices with greater clarity and spatial resolution.

The RANS model succeeds in identifying the principal vortex structures formed behind the vehicle ( Figure 6C), but it lacks accuracy in resolving their intensity, propagation, and dissipation downstream. This is attributed to the nature of the RANS approach, which models all turbulent scales via statistical averaging, limiting its ability to depict transient or fluctuating phenomena. In contrast, the DES model by blending RANS near walls with LES in detached regions captures a broader spectrum of turbulent scales ( Figure 6D). As a result, the DES simulation reveals a denser and more realistic distribution of vortical structures throughout the wake, particularly in the rotor and downstream zones.

Moreover, the application of the DES model clearly enhances the capture of both micro- and macro-scale vortices along the entire wake region. This improved resolution directly contributes to more accurate predictions of flow field variables, including the vehicle’s drag coefficient and streamline behaviour. The key differences in performance between these turbulence models arise from two fundamental features. First, the RANS model employs a statistical averaging method, decomposing flow variables into mean and fluctuating components, and is applied in a zonal fashion i.e., across the entire computational domain as predefined by the user. Second, the DES model operates as a wall-distance-based method. It dynamically transitions from RANS to LES depending on the distance of the mesh element from the wall and the local grid size, using a spatial filtering mechanism (∆ _LES_) and requiring mesh adaptation to function effectively.

### 3.4 Theoretical framework of vorticity dynamics

Figures 6A and 6B show the vortex structures in the near wake, revealing stretching and diffusion behaviour consistent with classical vorticity dynamics. In the RANS approach, small and medium turbulence scales are entirely modelled, so the analysis is limited to averaged large-scale vorticity structures. According to Bernard and Wallace, ^40^ the steady-state, averaged vorticity transport equation is expressed as:

$$\frac{D{\bar{\omega }}_{i}}{\mathrm{Dt}}={\bar{\omega }}_{j}\frac{\partial {\bar{u}}_{i}}{\partial x_{j}}+\bar{\omega \prime_{j}\frac{\mathrm{\partial u}\prime_{i}}{\partial x_{j}}}+\upsilon \frac{\partial^{2}{\bar{\omega }}_{i}}{\partial x_{j}^{2}}$$

**Equation 2.** Averaged vorticity transport equation.

Here, the left-hand side represents the convection of mean vorticity. The first term on the right-hand side corresponds to stretching due to the mean velocity field, the second term to stretching from turbulent fluctuations, and the third term to viscous diffusion. In RANS models using eddy-viscosity closures (such as Spalart–Allmaras), the fluctuating term is not directly resolved. Instead, the averaged vorticity field is derived from the velocity field obtained via the RANS equations.

This leads to a simplified form of the vorticity transport equation:

$$\frac{D{\bar{\omega }}_{i}}{\mathrm{Dt}}={\bar{\omega }}_{j}\frac{\partial {\bar{u}}_{i}}{\partial x_{j}}+\nabla \times \left(\nabla v_{t}\times \nabla \bar{u}\right)$$

**Equation 3.** Vorticity transport equation.

In this form, the two main physical mechanisms that influence the development of the mean vorticity field are stretching and eddy-viscosity-based $\bar{\omega }$ diffusion. The stretching term governs the rotation and amplification of vortical structures as they evolve downstream, while the diffusion term governs their spatial dispersion. This theoretical framework explains observed phenomena such as reconnection and cancellation of averaged vortical structures in the near wake.

### 3.5 3D Visualization of vortex structures

The generation and transport of vortex structures in the near and far wake of a vehicle can significantly affect its aerodynamic performance. These vortices, resulting from the interaction between the airflow and solid surfaces, are inherently three-dimensional phenomena. Therefore, visualising their evolution and intensity in 3D during post-processing is essential to identify optimisation strategies for vehicle components such as the chassis, fairing, or other elements influencing aerodynamic efficiency.

One widely used method for vortex visualisation is the Q-criterion, which is derived from the second invariant of the velocity gradient tensor, defined as:

$$Q=\frac{1}{2}\left({\left\|\Omega \right\|}^{2}-{\left\|S\right\|}^{2}\right)$$

**Equation 4**. Mathematical definition of the Q-criterion for vortex visualization.

Where S is the symmetric part of the velocity gradient tensor (the rate of strain), and Ω is the antisymmetric part (the vorticity tensor). A region where Q>0 indicates that the local vorticity magnitude dominates over the strain rate, and therefore represents the presence of a vortex. ^41^ Compared to planar vorticity iso-contours, the Q-criterion allows for a more detailed and three-dimensional visualisation of flow dynamics.

**Figure 7.**
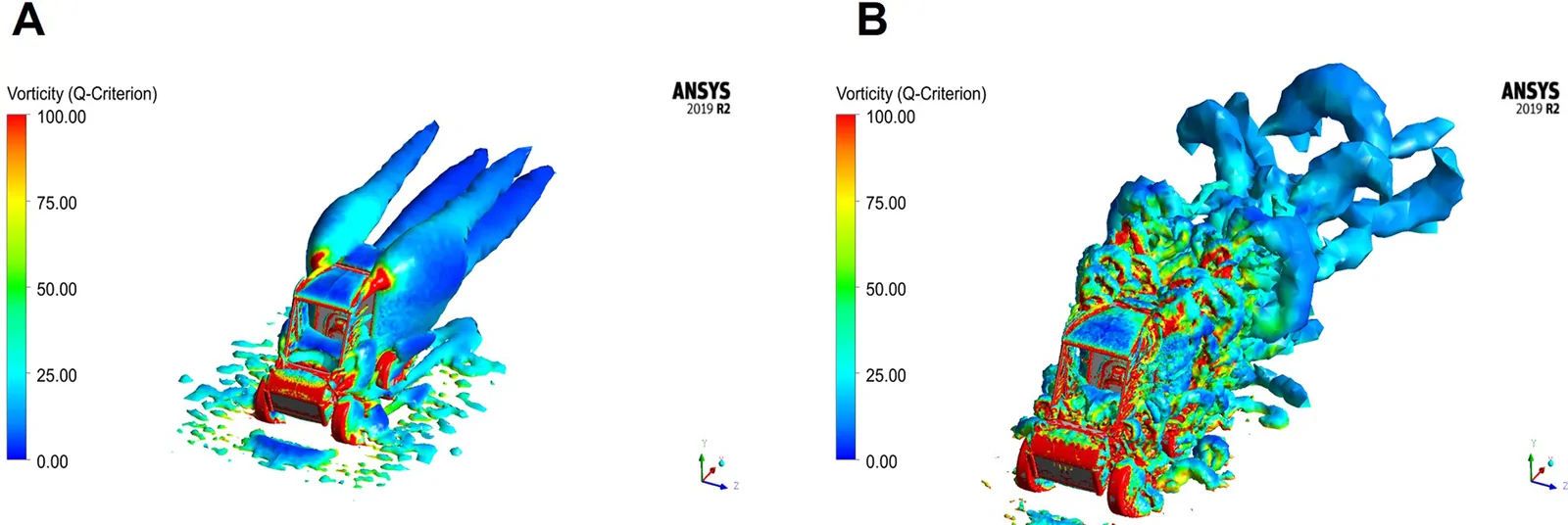
Isocontours of Q-criterion showing vortex structures around the vehicle and in the near wake region. A) RANS model colored by turbulence intensity; B) DES model colored by turbulence intensity.

Figure 7 presents a comparison of vortex structures obtained using the Q-criterion with both RANS (a) and DES (b) turbulence models. In both cases, the main large-scale vortex structures in the near and far wake of the vehicle are consistent with the aerodynamic principles proposed by Hucho. ^37^ These include counter-rotating vortices originating from the vertical uprights that extend from the roof towards the far wake, paired vortices resulting from the interaction between lateral and underbody flows, and small-scale vortices formed around the vehicle’s tires.

The key distinction between the two models lies in their ability to represent the evolution of vortex structures. While the RANS model captures the origin of large-scale vortices through a spatially averaged formulation, the DES model incorporates both spatial and temporal discretization, enabling a more detailed representation of unsteady flow features in the near wake. In particular, DES allows the identification of smaller-scale and secondary vortical structures that are not resolved under steady-state assumptions. Figure 7 further illustrates these differences through Q-criterion isocontours colored by turbulence intensity. Regions of high vorticity are observed around the front fairing, structural struts, and the frontal areas of the wheels locations where flow separation and vortex formation are expected. These features are consistent with the geometric characteristics of the Baja SAE vehicle, including flat frontal surfaces, exposed structural elements, and wheels extending beyond the chassis width, which are typical of off-road vehicle configurations.

The Q-criterion visualizations presented in this study are intended as qualitative indicators of vortex structures. Accordingly, these visualizations are intended to complement the drag coefficient analysis and should not be interpreted as a quantitative characterization of turbulence intensity or wake dynamics. A consistent threshold value was used across all cases; however, no sensitivity analysis of the isovalue was performed. Therefore, the comparison between RANS and DES should be interpreted in terms of general structural differences rather than as a quantitative assessment of vortex intensity.

The key distinction between the two models lies in their ability to resolve the evolution of these vortex structures. While the RANS model captures the origin of large-scale vortices and propagates them through a spatial averaging process, the DES model introduces both spatial and temporal discretisation. This enables more accurate resolution of both the location and evolution of vortical structures in the near wake, and allows for the capture of smaller, secondary vortices that RANS tends to neglect.

Figure 7 further illustrates these phenomena, showing isocontours of the Q-criterion colored by turbulence intensity. High-intensity vortices are observed at the front fairing, the struts, and the frontal areas of the front wheels regions where vortex formation initiates (RANS model in Figure 7A and DES model in Figure 7B). This behaviour is attributed to specific design characteristics of the Baja SAE vehicle: flat metal panels in the front fairing, uncovered structural struts formed from a single pipe frame integrated into the chassis, and wheels that extend beyond the width of the chassis, which is common in high performance of all-terrain vehicles (ATVs).

## 4. Discussion

The comparative analysis of turbulence models in this study provides relevant insights into the aerodynamic behavior of the Baja SAE vehicle, particularly in terms of wake dynamics and flow structure resolution. The drag coefficient (Cd) obtained using the RANS model was 1.290 ± 0.005, reflecting good numerical convergence and stability. In contrast, the hybrid RANS–LES (DES) model yielded a lower time-averaged Cd of 1.249 ± 0.024, capturing the inherent temporal variability associated with unsteady flow conditions. Rather than indicating improved predictive accuracy, this difference highlights the enhanced capability of DES to resolve transient turbulent structures, particularly in the wake region.

Flow visualizations further illustrate the differences between both modeling approaches. The RANS solution reveals two primary recirculation zones in the near wake, associated with flow inertia over the upper body and geometric discontinuities in the rear lower chassis. While these features are consistent with expected bluff-body behavior, the DES model provides a more detailed representation of low-velocity regions near the underbody and more complex flow patterns in the wake. In particular, alternating flow structures are observed downstream of the vehicle, suggesting the presence of unsteady vortex dynamics that are not captured under steady-state assumptions. These observations should be interpreted qualitatively, as the present study does not include spectral or frequency-based analyses required to rigorously characterize vortex shedding phenomena.

Streamline analysis of the RANS solution indicates the presence of counter-rotating flow structures in the near wake, consistent with patterns reported for squareback-type geometries. ^38, 40^ The vehicle configuration analyzed, with a rear slant angle of approximately 5°, exhibits flow behavior comparable to simplified Ahmed-body configurations, although the specific wake topology is influenced by the exposed structure and underbody geometry. It is important to note that, due to the steady-state nature of the RANS model, these streamline patterns represent time-averaged flow features and do not capture inherently unsteady wake dynamics.

The drag coefficient values obtained in this study (Cd ≈ 1.25–1.29) fall within the range typically reported for bluff-body and off-road vehicle configurations, supporting the physical plausibility of the numerical results. Nevertheless, the present study should not be interpreted as a validation of the aerodynamic performance of the Baja SAE vehicle. Instead, it should be regarded as a controlled numerical comparison between steady RANS and hybrid RANS–LES (DES) turbulence modeling strategies performed under identical computational conditions. Experimental validation through wind tunnel testing or on-track measurements is required before quantitative predictive capability can be established. Therefore, the conclusions presented here are restricted to the comparative behavior of the turbulence models and should not be interpreted as evidence of absolute aerodynamic prediction accuracy.

Although the present study does not include a parametric analysis, the identified flow features provide useful qualitative insights for aerodynamic design. The presence of strong recirculation zones and high-pressure regions in the rear and underbody suggests that modifications in rear geometry, improved surface continuity, and enhanced underbody flow management could contribute to drag reduction. Additionally, exposed structural elements and abrupt geometric transitions appear to promote flow separation, indicating potential benefits from streamlined fairing design. These observations can serve as a basis for future parametric and optimization studies.

It is also important to consider the limitations associated with the numerical setup. The use of stationary ground and non-rotating wheels represents a simplification of the actual operating conditions of a moving vehicle. These assumptions may alter the development of the underbody flow, wheel wake interactions, and the downstream wake structure, thereby influencing the quantitative prediction of aerodynamic forces. Nevertheless, because both turbulence models were evaluated under identical boundary conditions, the comparative assessment presented in this study remains internally consistent. Future work should incorporate moving-ground and rotating-wheel boundary conditions to improve the physical realism and predictive capability of the simulations.

Another limitation of the present numerical methodology is that mesh independence was formally assessed for the baseline RANS solution, whereas the DES mesh was constructed through local refinement following established hybrid RANS–LES recommendations. Although this approach provides an appropriate mesh for comparative analysis, a dedicated DES grid convergence study would further improve confidence in the quantitative prediction of unsteady flow structures. Such an analysis is planned as part of future work.

Finally, a more rigorous characterization of unsteady wake phenomena would require additional analyses such as Strouhal number estimation, spectral decomposition, or force coefficient frequency analysis, which were beyond the scope of this study. Future work should also incorporate experimental validation and parametric variations (e.g., velocity, yaw angle, and ride height) to achieve a more comprehensive aerodynamic assessment.

## 5. Conclusion

This study presents a comparative aerodynamic analysis of a Baja SAE vehicle using RANS and hybrid RANS–LES (DES) turbulence models. While the RANS approach provided stable and computationally efficient results, it showed limitations in resolving vortex dynamics and turbulent fluctuations in the wake. In contrast, the DES model captured a broader spectrum of vortex structures, particularly in the near and far wake, enabling a more detailed representation of unsteady flow behaviour. The DES approach predicted a lower time-averaged drag coefficient (1.249 ± 0.024) compared to RANS (1.290 ± 0.005), and provided enhanced spatial resolution of velocity, pressure, and vorticity fields. Q-criterion and streamline visualizations further supported its capability to represent multi-scale turbulent structures, offering deeper insight into wake dynamics and their influence on aerodynamic performance. The drag coefficient values obtained (Cd ≈ 1.25–1.29) are consistent with those reported for bluff-body and off-road vehicle configurations, supporting the physical plausibility of the results. However, in the absence of experimental validation, the findings should be interpreted as a comparative assessment of turbulence modeling approaches rather than as a fully validated aerodynamic prediction.

Although the study is limited to a single geometry and operating condition, the identified flow features provide useful qualitative insights for aerodynamic design, particularly regarding the influence of rear geometry, surface discontinuities, and underbody flow on wake formation and drag generation. The present work also involves several modeling simplifications, including the use of stationary ground and non-rotating wheels, which may affect near-ground flow development and the absolute values of aerodynamic forces. Nevertheless, since both RANS and DES simulations were performed under identical conditions, the comparative analysis remains valid.

Overall, the results highlight the potential of hybrid RANS–LES methods as a valuable tool for the comparative analysis of complex bluff-body flows. Because the present investigation was intentionally limited to a single vehicle geometry and one operating condition, the conclusions should be interpreted as a baseline comparison of turbulence modeling approaches rather than as a comprehensive aerodynamic assessment. Future work will focus on experimental validation, dedicated DES grid convergence analysis, incorporation of moving ground and rotating wheels, and broader parametric investigations including velocity, yaw angle, and ride height, in order to improve predictive fidelity, evaluate the generality of the observed trends, and support aerodynamic design optimization.

## Use of artificial intelligence

We acknowledge that artificial intelligence tools (ChatGPT, OpenAI) were employed exclusively to assist with the revision of the English language and to improve the clarity of the writing. All scientific content, analyses, and conclusions are entirely the work of the authors.

## Data Availability

The materials supporting the findings of this study are available in the Zenodo repository: https://doi.org/10.5281/zenodo.17295975. ^43^ These materials include the ANSYS simulation files used to generate the computational results presented in the paper (geometry, mesh, boundary conditions, and solver configuration). No external datasets were used in this study, as all results derive from numerical simulations developed by the authors. The files are shared under a Creative Commons Attribution 4.0 International (CC BY 4.0) license, allowing reuse and adaptation with proper citation. Due to file size limitations, the complete raw simulation output can be provided upon reasonable request to the corresponding author. Additional materials supporting this study are available in the Zenodo repository: DOI: https://doi.org/10.5281/zenodo.17295975. ^43^ This record includes the ANSYS project files used in the numerical simulations described in the manuscript, comprising the 3D geometry model of the vehicle, computational mesh file, boundary condition setup, solver configuration, and post-processing parameters. All files are shared under a Creative Commons Attribution 4.0 International (CC BY 4.0) license.
