# Supplementary material for: Aerodynamic Performance of a Baja SAE Vehicle Using Hybrid RANS-LES Approach
Source: F1000Res. 2026 Jul 25;14:1199. Originally published 2025 Nov 3. [Version 3] doi: 10.12688/f1000research.171076.3 (PMC13560008; doi:10.12688/f1000research.171076.3)
Supplement: Peer review history

# Reviewer report: Reviewer response for version 3

**DOI:** 10.5256/f1000research.206697.r505518

**Published:** 2026-08-20

**Authors:** Sheharyar Nasir

**Affiliations:** 1 The University of Kansas, Lawrence, Kansas, USA

**Overall Report**

 The manuscript presents a computational investigation of the aerodynamic performance of a Baja SAE vehicle using steady RANS and hybrid RANS–LES through the Detached Eddy Simulation (DES) approach. The study compares the two modeling approaches primarily through drag coefficient predictions and analysis of the resulting pressure, velocity, vorticity, and wake structures. The topic is relevant to computational vehicle aerodynamics, particularly in demonstrating how different turbulence modeling approaches represent separated and unsteady wake flows.

 I have reviewed the latest version of the manuscript and considered the revisions made during the previous review rounds. In my assessment, the manuscript has improved considerably. The objective of the study is now more clearly defined, the numerical methodology is better explained, and the limitations of the computational analysis are presented more transparently. Importantly, the work is now more appropriately framed as a comparative numerical study of RANS and DES, rather than as a fully validated prediction of the vehicle's aerodynamic performance. This distinction is scientifically important given the absence of direct experimental validation.

 Within this defined scope, I find the study design appropriate and the work technically sound. The comparison is performed under consistent computational conditions, and the results provide a useful demonstration of the differences between steady RANS and DES in representing the vehicle wake. The revised discussion also provides a more balanced interpretation of the reported drag coefficients. In particular, the manuscript now recognizes that the difference between the RANS and DES values should not itself be interpreted as evidence that DES provides a more accurate prediction of the actual vehicle drag.

 I also find the conclusions generally supported by the numerical results, provided they remain within this comparative framework. DES resolves additional transient and smaller-scale structures in the wake that cannot be represented in the same manner by the steady RANS solution. However, without experimental or other validated reference data, improved resolution of these structures should remain clearly distinguished from improved predictive accuracy.

 My main remaining concern is reproducibility. Although the numerical methodology is described in reasonable detail, I selected Partly **,** regarding whether sufficient methodological information is provided to allow complete replication by another researcher. This concern is particularly relevant to the DES calculation. The authors acknowledge that the mesh-independence assessment was conducted for the baseline RANS solution and that a dedicated DES grid-convergence analysis was not performed. Because DES behavior is sensitive to local grid resolution, more complete documentation of the computational setup would improve reproducibility.

 I do not consider an additional full DES grid-convergence study necessary to establish the basic scientific validity of the present comparative investigation. However, the authors should ensure that the final manuscript or supplementary information clearly provides the practical information required to reconstruct the DES calculation. In particular, the local refinement strategy and relevant cell sizes, timestep and temporal discretization, sampling and averaging interval used to obtain the reported DES drag coefficient, and important solver settings should be stated clearly. A dedicated DES mesh-sensitivity investigation would then be a valuable extension in future work.

 I also selected Partly regarding the availability of all source data required for full reproducibility. The provision of extended data is appreciated, and the manuscript identifies an associated extended-data repository. Nevertheless, for a computational CFD study, reproducibility requires more than the final numerical results. Another researcher should have sufficient information to reconstruct the computational configuration that generated those results. I therefore recommend that the authors clearly state what computational files and data are available and ensure that the repository or supplementary information contains, as far as reasonably possible, the geometry/mesh information, numerical setup, boundary conditions, DES refinement information, and definitions used in post-processing. If certain proprietary ANSYS Fluent files cannot be shared, the corresponding settings should at least be documented sufficiently to permit reconstruction of an equivalent simulation.

 The absence of experimental validation remains an important limitation, but I do not consider it a fundamental flaw in the present manuscript because the authors now acknowledge it and appropriately restrict the scope of their conclusions. The comparison with published aerodynamic data provides useful context for assessing whether the calculated drag coefficients are physically plausible, but such comparison should continue to be described as benchmarking rather than validation. The current manuscript makes this distinction considerably better than the earlier versions.

 Similarly, the assumptions of stationary ground and non-rotating wheels, the consideration of only one geometry and operating condition, and the focus primarily on drag coefficient limit the extent to which the results can be generalized to the complete aerodynamic behavior of a Baja SAE vehicle. However, these limitations are now clearly identified by the authors, and the manuscript does not need to be expanded into a complete vehicle-aerodynamics study to support its present objective. Moving ground, rotating wheels, additional force and moment coefficients, different operating conditions, and quantitative wake diagnostics would be appropriate extensions for future work rather than requirements for the current comparative investigation.

 Overall, I find that the manuscript has reached a scientifically acceptable form within its stated scope. The numerical comparison between RANS and DES is useful, the methodology is generally appropriate, and the conclusions are now presented with considerably better recognition of the limitations of the simulations. My remaining comments primarily concern reproducibility and precision in the interpretation of DES rather than fundamental problems with the study.

 Before final acceptance, I recommend that the authors address the following points:

1. Review the manuscript for any remaining statements implying that DES has been demonstrated to provide greater predictive accuracy. Where appropriate, these should instead refer to improved temporal or spatial resolution of the unsteady wake unless supported by validation data.
2. Provide or clarify the numerical information necessary to reproduce the DES simulation, particularly the mesh/refinement characteristics, timestep, temporal sampling and averaging procedure, and relevant solver settings.
3. Clearly describe what computational source data and setup information are available in the associated repository or supplementary material so that the level of reproducibility can be assessed by future researchers.

These points can be addressed through clarification and improved documentation and, in my view, do not require major additional simulations.

Is the work clearly and accurately presented and does it cite the current literature?

Yes

If applicable, is the statistical analysis and its interpretation appropriate?

Not applicable

Are all the source data underlying the results available to ensure full reproducibility?

Partly

Is the study design appropriate and is the work technically sound?

Yes

Are the conclusions drawn adequately supported by the results?

Yes

Are sufficient details of methods and analysis provided to allow replication by others?

Partly

Reviewer Expertise:

Computational Fluid Dynamics (CFD), Aerodynamics, Turbulence and Turbulence Modeling, Computational Combustion, Reacting Flows, Reduced-Order Modeling, Data-Driven Modeling.

I confirm that I have read this submission and believe that I have an appropriate level of expertise to confirm that it is of an acceptable scientific standard.

# Reviewer report: Reviewer response for version 3

**DOI:** 10.5256/f1000research.206697.r504769

**Published:** 2026-08-08

**Authors:** Luis Fernando Rodriguez

**Affiliations:** 1 Mechanical and Aerospace Engineering, Clarkson University, Potsdam, New York, USA

**General Assessment**

 I appreciate the considerable effort the authors have invested in revising the manuscript. Compared with the previous versions, the paper has undergone substantial improvements in scientific rigor, methodological transparency, and presentation. The authors have carefully considered the comments raised during the previous review rounds and have addressed the majority of the concerns either through manuscript revisions or by explicitly acknowledging the remaining limitations of the study.

 The manuscript is now more accurately presented as a comparative computational investigation of turbulence modeling approaches for Baja SAE aerodynamics rather than as a fully validated aerodynamic prediction. This revised positioning is scientifically appropriate and considerably strengthens the credibility of the work.

 Overall, the manuscript has matured significantly and now provides a coherent and technically sound contribution within the scope defined by the authors.

**Assessment of Previous Review Comments**

**1. Experimental Validation**

*Previous concern:* The original manuscript lacked any form of experimental validation or comparison with reliable benchmark data.

*Assessment:* Partially addressed. Experimental validation has not been incorporated and remains the principal limitation of the work. However, the authors now explicitly acknowledge this limitation and appropriately frame the study as a numerical comparative investigation. Comparisons with published literature provide useful context.

 Status: **Acceptable**.

**2. Scope of the Aerodynamic Analysis**

*Previous concern:* The manuscript focused almost exclusively on drag coefficient.

*Assessment:* Adequately addressed. The authors clearly justify that the objective is to compare turbulence models rather than provide a complete aerodynamic characterization.

 Status: **Resolved**.

**3. Detached Eddy Simulation (DES) Methodology**

*Previous concern*: The DES implementation and filter-size definition required clarification.

*Assessment*: Well - addressed. The methodology has been clarified, and the previous ambiguity has been removed. Although a DES specific mesh convergence study would further strengthen the work, the current description is technically satisfactory.

 Status: **Resolved**.

**4. Interpretation of Flow Structures**

*Previous concern*: Excessive reliance on qualitative flow visualizations.

*Assessment*: Substantially improved. The authors now distinguish qualitative observations from quantitative conclusions and avoid overinterpreting the numerical results.

 Status: **Resolved**.

**5. Numerical Assumptions**

*Previous concern*: Stationary ground and non-rotating wheels reduced physical realism.

*Assessment*: Adequately addressed. These assumptions are now explicitly discussed and recognized as limitations of the study.

 Status: **Resolved**.

**6. Scope of the Investigation**

*Previous concern*: Only one geometry and one operating condition were investigated.

*Assessment*: Acceptably addressed. The authors clearly define the study as a baseline computational investigation and avoid unsupported generalizations.

 Status: **Acceptable**.

**7. Overall Scientific Interpretation**

*Assessment*: One of the strongest improvements is the balanced interpretation of the numerical results. The revised discussion appropriately distinguishes computational observations from experimentally validated conclusions.

**Remaining Minor Comments**

 1. A brief statement emphasizing future experimental validation could be added to the Conclusions.

 2. Figure captions could include additional quantitative information.

 3. Minor grammatical polishing would further improve readability.

 4. A dedicated DES mesh convergence study is recommended for future work.

 Final Recommendation: **Approved.**

 The revised manuscript satisfactorily addresses the majority of the concerns raised during the previous review rounds. Although certain limitations remain (notably the absence of experimental validation and the limited scope of the aerodynamic characterization) the authors now acknowledge these limitations explicitly and present their conclusions within an appropriate scientific framework.

 The manuscript represents a significant improvement over the original submission and now provides a technically sound comparative study of RANS and DES methodologies applied to Baja SAE vehicle aerodynamics.

 The remaining limitations do not invalidate the scientific contribution of the work and can be addressed in future investigations.

**Final Comment to the Authors**

 I congratulate the authors on the substantial improvements made throughout the review process. The manuscript demonstrates clear responsiveness to reviewer feedback and now presents a much stronger and more scientifically balanced contribution.

 Future work incorporating experimental validation, moving ground simulations, rotating wheels, additional aerodynamic force coefficients, and quantitative wake analyses would further enhance the impact and applicability of this research.

Is the work clearly and accurately presented and does it cite the current literature?

Partly

If applicable, is the statistical analysis and its interpretation appropriate?

Partly

Are all the source data underlying the results available to ensure full reproducibility?

Partly

Is the study design appropriate and is the work technically sound?

Partly

Are the conclusions drawn adequately supported by the results?

Partly

Are sufficient details of methods and analysis provided to allow replication by others?

Partly

Reviewer Expertise:

Mechanical Engineering: Structural Analysis and Fluid Mechanics (Turbulent Analysis).

I confirm that I have read this submission and believe that I have an appropriate level of expertise to confirm that it is of an acceptable scientific standard.

# Reviewer report: Reviewer response for version 2

**DOI:** 10.5256/f1000research.203182.r493250

**Published:** 2026-06-26

**Authors:** Luis Fernando Rodriguez

**Affiliations:** 1 Mechanical and Aerospace Engineering, Clarkson University, Potsdam, New York, USA

The revised manuscript demonstrates noticeable improvement in clarity, scope definition, and acknowledgment of methodological limitations. The authors have responded carefully to the first-round comments and corrected several conceptual weaknesses.

 However, despite these improvements, the manuscript still presents important scientific limitations that restrict its impact and technical rigor. While the revised version is significantly stronger, several critical issues remain only partially addressed.

*Assessment of Major Review Points:*

*1. Validation and Benchmarking Assessment:* Partially addressed, but still insufficient. The inclusion of literature benchmarking improves the contextualization of the drag coefficient values; however, benchmarking is not equivalent to validation. No experimental, wind tunnel, or track data are presented. Therefore, the predictive capability of both RANS and DES remains unverified. This continues to be the most important weakness of the work.

*2. Scope of Aerodynamic Metrics Assessment:* Only partially resolved. The authors justify focusing exclusively on drag coefficient, but for a racing/off-road vehicle this remains a narrow representation of aerodynamic performance. The absence of lift, side force, and aerodynamic moments significantly limits practical applicability. Although acceptable for a methodological study, this omission reduces engineering relevance.

*3. DES Methodological Justification Assessment:* Improved but still incomplete. The correction of the LES filter size formulation resolves the earlier circular reasoning issue. However, the lack of a DES specific grid convergence analysis remains a methodological weakness. Since DES is highly mesh dependent, RANS based mesh independence is not sufficient to guarantee DES fidelity.

*4. Flow Visualization Interpretation Assessment*: Improved. The authors appropriately softened claims regarding vortex shedding and now treat Q criterion and vorticity results qualitatively. This is a positive correction. However, the manuscript still relies heavily on visualization without sufficient quantitative support.

*5. Boundary Condition Simplifications Assessment*: Acknowledged but not resolved. The discussion regarding stationary ground and non-rotating wheels is clearer, but the physical impact of these assumptions remains substantial. For vehicle aerodynamics, especially wake studies, this simplification can alter the flow field significantly. This limitation weakens the realism of the results.

*6. Parametric Scope Assessment:* Weakly addressed. The manuscript still investigates only one operating condition and one geometry. While the authors now define this as a baseline study, the scientific contribution remains limited because no broader aerodynamic insight or optimization trend can be extracted.

*7. Quantitative Wake Diagnostics Assessment:* Still insufficient. The absence of spectral analysis, Strouhal number estimation, or force frequency decomposition prevents rigorous identification of wake dynamics. The authors correctly acknowledge this limitation, but its absence still weakens the depth of the study.

*Remaining Weaknesses*

 1. No direct validation of drag or wake structures.

 2. No DES specific mesh convergence study.

 3. No lift, side force, or aerodynamic moments. 4. Strong dependence on qualitative visualizations.

 5. Simplified ground and wheel modeling reduce realism.

 6. Single case scope limits broader contribution.

*Final Recommendation:*

**Major Revision / Borderline Acceptance.**

 The manuscript is substantially improved compared to Version 1 and now demonstrates better scientific honesty and methodological clarity. However, from an archival indexing perspective, the absence of validation and the limited quantitative depth remain significant concerns.

 The study is acceptable as an exploratory computational comparison, but it still falls short of the rigor expected for a high impact aerodynamic journal.

*Final Comment to the Authors*

 The revisions clearly improve the manuscript and show strong responsiveness to the reviewer feedback. The work has educational and methodological value, particularly for Baja SAE applications.

 However, to reach a stronger scientific standard, future work must include experimental validation, moving ground simulations, rotating wheels, expanded force analysis, and quantitative wake diagnostics. These additions would transform the manuscript from a comparative CFD exercise into a robust aerodynamic contribution.

Is the work clearly and accurately presented and does it cite the current literature?

Partly

If applicable, is the statistical analysis and its interpretation appropriate?

Partly

Are all the source data underlying the results available to ensure full reproducibility?

Partly

Is the study design appropriate and is the work technically sound?

Partly

Are the conclusions drawn adequately supported by the results?

Partly

Are sufficient details of methods and analysis provided to allow replication by others?

Partly

Reviewer Expertise:

Mechanical Engineering: Structural Analysis and Fluid Mechanics (Turbulent Analysis).

I confirm that I have read this submission and believe that I have an appropriate level of expertise to confirm that it is of an acceptable scientific standard, however I have significant reservations, as outlined above.

# Response

**Published:** 2026-07-17

**Authors:** Deisy Yurley Rodriguez Sarmiento

**Affiliations:** Universidad Autonoma de Bucaramanga, Bucaramanga, Santander, Colombia

*1. Validation and Benchmarking Assessment:* Partially addressed, but still insufficient.

 The inclusion of literature benchmarking improves the contextualization of the drag coefficient values; however, benchmarking is not equivalent to validation. No experimental, wind tunnel, or track data are presented. Therefore, the predictive capability of both RANS and DES remains unverified. This continues to be the most important weakness of the work.

 Response: We sincerely thank the reviewer for this important observation and fully agree that benchmarking against published drag coefficient ranges cannot replace experimental validation. The absence of wind tunnel, track, or other experimental measurements remains a limitation of the present work.

 However, the primary objective of this study is not to establish a fully validated aerodynamic model of the Baja SAE vehicle, but rather to perform a comparative evaluation of steady RANS and hybrid RANS–LES (DES) turbulence modeling approaches under identical numerical conditions. Accordingly, the manuscript has been carefully revised to avoid claims regarding predictive accuracy and to explicitly state that the results should be interpreted as a comparative numerical assessment rather than a validated aerodynamic prediction.

 To further emphasize this point, we have strengthened the Discussion and Conclusions by explicitly indicating that the predictive capability of the simulations remains unverified in the absence of experimental data, and that future work will focus on wind tunnel or on-track validation together with more realistic boundary conditions (moving ground and rotating wheels).

 We believe that these revisions appropriately define the scope of the study while maintaining scientific transparency regarding its limitations. (Line 443)

*2. Scope of Aerodynamic Metrics Assessment:* Only partially resolved.

 The authors justify focusing exclusively on drag coefficient, but for a racing/off-road vehicle this remains a narrow representation of aerodynamic performance. The absence of lift, side force, and aerodynamic moments significantly limits practical applicability. Although acceptable for a methodological study, this omission reduces engineering relevance.

 Response: We thank the reviewer for this valuable observation and agree that a comprehensive aerodynamic characterization of a ground vehicle ideally includes lift, side force, and aerodynamic moments. These quantities are indeed important for evaluating vehicle stability and handling performance.

 However, the primary objective of the present work is not to provide a complete aerodynamic characterization of the Baja SAE vehicle, but rather to compare the performance of steady RANS and hybrid RANS–LES (DES) turbulence modeling approaches in resolving wake flow structures under identical computational conditions. For this reason, the analysis focuses on the drag coefficient, which is directly associated with wake-induced aerodynamic losses and provides an appropriate metric for evaluating the influence of the turbulence model on flow separation and vortex dynamics.

 To avoid any ambiguity regarding the scope of the study, the manuscript explicitly states that the analysis is limited to drag and does not constitute a complete aerodynamic force and moment assessment. We have further reinforced this clarification in the Discussion and Conclusion sections and identified the evaluation of lift, side force, and aerodynamic moments as part of future work.(Line 117)

*3. DES Methodological Justification Assessment:* Improved but still incomplete.

 The correction of the LES filter size formulation resolves the earlier circular reasoning issue. However, the lack of a DES specific grid convergence analysis remains a methodological weakness. Since DES is highly mesh dependent, RANS based mesh independence is not sufficient to guarantee DES fidelity.

 Response: We thank the reviewer for this important observation and fully agree that hybrid RANS–LES methods are inherently more sensitive to grid resolution than steady RANS simulations, and that a dedicated DES grid convergence study would further strengthen the numerical assessment.

 In the present work, the mesh independence study was performed using the drag coefficient as the convergence criterion for the baseline RANS simulation, while the DES mesh was subsequently refined according to established DES guidelines, including local wake refinement based on the estimated LES filter size and the corresponding grid resolution required for resolving detached turbulent structures.

 The objective of this study was not to establish formal DES grid convergence, but rather to compare the behavior of RANS and DES under computationally consistent conditions. We therefore acknowledge that the absence of a dedicated DES grid convergence analysis constitutes a limitation of the present work and have explicitly included this point in the Discussion as a direction for future research. (Line 468)

*4. Flow Visualization Interpretation Assessment*: Improved.

 The authors appropriately softened claims regarding vortex shedding and now treat Q criterion and vorticity results qualitatively. This is a positive correction. However, the manuscript still relies heavily on visualization without sufficient quantitative support.

 Response: We appreciate the reviewer's observation and agree that quantitative wake diagnostics would provide a more comprehensive characterization of the flow field.

 The primary objective of the present study, however, is to compare the qualitative capability of steady RANS and hybrid RANS–LES (DES) turbulence models to represent wake structures under identical computational conditions, rather than to perform a complete quantitative turbulence characterization.

 Accordingly, the manuscript has been revised to consistently present the Q-criterion, vorticity contours, and flow visualizations as qualitative indicators of wake behavior, while avoiding interpretations beyond the available numerical evidence.

 We further acknowledge in the Discussion that additional quantitative analyses, including spectral decomposition, Strouhal number estimation, turbulence statistics, and force frequency analysis, would provide valuable complementary information and are planned for future work.(line 406)

*5. Boundary Condition Simplifications Assessment*: Acknowledged but not resolved.

 The discussion regarding stationary ground and non-rotating wheels is clearer, but the physical impact of these assumptions remains substantial. For vehicle aerodynamics, especially wake studies, this simplification can alter the flow field significantly. This limitation weakens the realism of the results.

 Response: We thank the reviewer for this important observation and agree that the use of a stationary ground and non-rotating wheels represents a significant simplification of the real operating conditions of a ground vehicle. These assumptions may influence the development of the underbody flow, wheel wake interactions, and the overall wake structure, thereby affecting the quantitative prediction of aerodynamic forces.

 However, the primary objective of this work is to compare the relative performance of steady RANS and hybrid RANS–LES (DES) turbulence models under identical computational conditions rather than to reproduce the complete aerodynamic behavior of the vehicle under real driving conditions. Since both turbulence models were evaluated using the same boundary conditions, the comparative assessment remains internally consistent.

 To further clarify this point, the Discussion has been strengthened to explicitly state that these boundary-condition simplifications limit the quantitative realism of the results and that future work will incorporate moving-ground and rotating-wheel models to improve aerodynamic fidelity.

*6. Parametric Scope Assessment:* Weakly addressed.

 The manuscript still investigates only one operating condition and one geometry. While the authors now define this as a baseline study, the scientific contribution remains limited because no broader aerodynamic insight or optimization trend can be extracted.

 Response: We thank the reviewer for this thoughtful comment and agree that a broader parametric investigation involving multiple operating conditions, vehicle geometries, or aerodynamic optimization strategies would provide additional engineering insight.

 However, the objective of the present work is intentionally more focused. The study was designed as a baseline numerical investigation to isolate and compare the performance of steady RANS and hybrid RANS–LES (DES) turbulence models under a single, controlled operating condition. Restricting the analysis to one geometry and one flow condition minimizes additional variables that could obscure the influence of the turbulence modeling approach.

 We have further clarified in the manuscript that the conclusions are limited to this comparative framework and should not be interpreted as a generalized aerodynamic assessment of Baja SAE vehicles. We also identify broader parametric studies involving different operating conditions, vehicle configurations, and aerodynamic optimization as important directions for future research.

*7. Quantitative Wake Diagnostics Assessment:* Still insufficient.

 The absence of spectral analysis, Strouhal number estimation, or force frequency decomposition prevents rigorous identification of wake dynamics. The authors correctly acknowledge this limitation, but its absence still weakens the depth of the study.

 Response: We thank the reviewer for this valuable observation and agree that quantitative wake diagnostics, such as spectral analysis, Strouhal number estimation, and force frequency decomposition, would provide a more rigorous characterization of the unsteady wake dynamics. These analyses are particularly valuable for identifying dominant flow frequencies and quantifying vortex shedding phenomena.

 However, the objective of the present work is to compare the performance of steady RANS and hybrid RANS–LES (DES) turbulence models under identical computational conditions rather than to perform a comprehensive investigation of wake dynamics. Accordingly, the flow visualizations are presented as qualitative evidence supporting the observed differences in drag prediction.

 As recommended by the reviewer, the manuscript explicitly acknowledges that the absence of quantitative wake diagnostics constitutes a limitation of the present study, and future work will incorporate spectral analyses and frequency-based metrics to provide a more complete characterization of the unsteady flow.

*Remaining Weaknesses*

 1. No direct validation of drag or wake structures.

 2. No DES specific mesh convergence study.

 3. No lift, side force, or aerodynamic moments. 4. Strong dependence on qualitative visualizations.

 5. Simplified ground and wheel modeling reduce realism.

 6. Single case scope limits broader contribution.

 Response: We sincerely thank the reviewer for the careful evaluation and constructive suggestions provided throughout the review process. We acknowledge that the present study retains several limitations, including the absence of direct experimental validation, a dedicated DES grid convergence study, additional aerodynamic force and moment analyses, comprehensive quantitative wake diagnostics, more realistic moving-ground and rotating-wheel boundary conditions, and a broader parametric investigation.

 These aspects have now been explicitly recognized and discussed as limitations in the revised manuscript. However, we respectfully emphasize that the primary objective of this work is to provide a controlled comparative assessment of steady RANS and hybrid RANS–LES (DES) turbulence models under identical computational conditions, rather than a complete aerodynamic characterization or optimization study. We believe that the revisions have clarified the scope, strengthened the discussion of the methodological limitations, and improved the scientific rigor and transparency of the manuscript.

# Reviewer report: Reviewer response for version 1

**DOI:** 10.5256/f1000research.188621.r469214

**Published:** 2026-04-10

**Authors:** Luis Fernando Rodriguez

**Affiliations:** 1 Mechanical and Aerospace Engineering, Clarkson University, Potsdam, New York, USA

General Assessment

 The manuscript presents an interesting computational study of the aerodynamic performance of a Baja SAE vehicle using both RANS and hybrid RANS–LES (DES) turbulence models. The authors demonstrate strong technical effort and familiarity with advanced CFD tools. The work has clear educational value and provides useful insight into the application of hybrid turbulence modeling for vehicle aerodynamics.

 Strengths

 The study includes a well-developed CFD methodology, a detailed meshing strategy, and a thoughtful comparison between RANS and DES approaches. The use of post-processing techniques such as vorticity contours and Q-criterion visualization is appropriate and helps illustrate key flow features. The manuscript is also well motivated within the context of Baja SAE design.

 Points for Improvement

 1. Validation: The manuscript would benefit significantly from validation against experimental data, simplified benchmarks, or published results. Including such comparisons would strengthen the credibility of the conclusions.

 2. Aerodynamic Metrics: Expanding the analysis beyond drag coefficient to include lift, side forces, or moments would provide a more complete characterization of vehicle performance.

 3. Turbulence Model Justification: Additional discussion on why DES is necessary for this flow regime, including comparisons with simpler models, would improve the methodological clarity.

 4. Boundary Conditions: The assumption of stationary ground and non-rotating wheels should be discussed in more detail, including its potential impact on results.

 5. Quantitative Analysis: Complementing visualizations with quantitative metrics (e.g., frequency analysis or additional flow parameters) would strengthen the analysis.

 6. Scope: Exploring additional cases (such as different speeds or geometric variations) could enhance the practical impact of the work.

 Minor Comments

 The introduction could more clearly define the research gap. Some figures would benefit from additional quantitative annotations. The organization of the methods section could also be improved for clarity.

 Recommendation

 The manuscript has solid potential but would benefit from revisions addressing the points above. With additional validation, expanded analysis, and clearer justification of modeling choices, the work could make a valuable contribution to the field.

 Final Comment

 Overall, this is a promising study that demonstrates strong technical effort. With further refinement and strengthening of the scientific rigor, it could be suitable for indexing.

Is the work clearly and accurately presented and does it cite the current literature?

Partly

If applicable, is the statistical analysis and its interpretation appropriate?

Partly

Are all the source data underlying the results available to ensure full reproducibility?

Partly

Is the study design appropriate and is the work technically sound?

Partly

Are the conclusions drawn adequately supported by the results?

Partly

Are sufficient details of methods and analysis provided to allow replication by others?

Partly

Reviewer Expertise:

Mechanical Engineering: Structural Analysis and Fluid Mechanics (Turbulent Analysis).

I confirm that I have read this submission and believe that I have an appropriate level of expertise to state that I do not consider it to be of an acceptable scientific standard, for reasons outlined above.

# Response

**Published:** 2026-04-21

**Authors:** Deisy Yurley Rodriguez Sarmiento

**Affiliations:** Universidad Autonoma de Bucaramanga, Bucaramanga, Santander, Colombia

Points for Improvement

  

1. Validation: The manuscript would benefit significantly from validation against experimental data, simplified benchmarks, or published results. Including such comparisons would strengthen the credibility of the conclusions.

Response: We thank the reviewer for this valuable suggestion. The importance of validation is fully acknowledged and was also raised by Reviewer 1. In response, we have revised the manuscript to clarify that the present work is a comparative numerical study of turbulence modeling approaches rather than a fully validated aerodynamic prediction.

 Additionally, we have included a benchmarking discussion showing that the predicted drag coefficient values fall within ranges reported in the literature for similar bluff-body and off-road vehicle configurations, supporting the physical plausibility of the results.

 The absence of experimental validation is now explicitly acknowledged as a limitation, and future work will address this through experimental and parametric studies.

  

1. Aerodynamic Metrics: Expanding the analysis beyond drag coefficient to include lift, side forces, or moments would provide a more complete characterization of vehicle performance.

Response: We thank the reviewer for this valuable suggestion. This point was also raised by Reviewer 1 and has been addressed in the revised manuscript. We acknowledge that a complete aerodynamic characterization would include additional force components such as lift, side force, and aerodynamic moments. However, the objective of the present study is to provide a comparative assessment of turbulence modeling approaches, focusing on wake dynamics and drag as a representative parameter of wake-induced aerodynamic losses. This scope has now been clearly stated in the revised manuscript.  Additionally, we have included a statement highlighting that future work will incorporate a more comprehensive set of aerodynamic metrics to enable a complete evaluation of vehicle performance.

  

1. Turbulence Model Justification: Additional discussion on why DES is necessary for this flow regime, including comparisons with simpler models, would improve the methodological clarity.

Response: This aspect was also raised by Reviewer 1 and has been addressed in the revised manuscript. We clarify that, although the Reynolds number considered falls within a range where steady RANS approaches can provide reasonable predictions of global aerodynamic quantities, the use of DES is motivated by its ability to better represent unsteady flow structures, particularly in the wake region. The manuscript has been revised to explicitly state that the use of DES is intended to enhance the representation of wake dynamics rather than to serve as a strictly necessary modeling approach for this flow regime. Also, the scope of the study has been clarified as a comparative evaluation of turbulence modeling approaches, rather than a fully optimized or validated aerodynamic prediction.

  

1. Boundary Conditions: The assumption of stationary ground and non-rotating wheels should be discussed in more detail, including its potential impact on results.

Response: This aspect was also raised by Reviewer 1 and has been addressed in the revised manuscript. We have included a more detailed discussion of the assumption of stationary ground and non-rotating wheels, highlighting its potential impact on near-ground flow development, wake structure, and the absolute values of aerodynamic forces. This modeling choice has been clarified as a simplification adopted to maintain computational feasibility and ensure consistency in the comparative analysis between turbulence models.

 The corresponding limitations are now explicitly stated in both the Methods and Discussion sections, and future work will incorporate moving ground conditions and rotating wheels to improve the physical realism of the simulations.

  

1. Quantitative Analysis: Complementing visualizations with quantitative metrics (e.g., frequency analysis or additional flow parameters) would strengthen the analysis.

Response: This point was also raised by Reviewer 1 and has been addressed in the revised manuscript. We clarify that the flow visualizations presented in this study are intended for qualitative analysis of flow structures rather than for quantitative characterization of unsteady phenomena. Accordingly, we have revised the manuscript to avoid overinterpretation and to explicitly state the limitations of the visual analysis. We also acknowledge that a more rigorous quantitative characterization such as frequency analysis, Strouhal number estimation, or additional flow metrics would further strengthen the analysis, but is beyond the scope of the present study. Future work will incorporate these approaches to provide a more comprehensive assessment of unsteady wake dynamics.

  

1. Scope: Exploring additional cases (such as different speeds or geometric variations) could enhance the practical impact of the work.

Response: This aspect was also raised by Reviewer 1 and has been addressed in the revised manuscript. We acknowledge that the present study is limited to a single geometry and operating condition, and therefore does not capture the influence of parameters such as velocity or geometric variations.

 The scope of the work has been clarified as a comparative evaluation of turbulence modeling approaches rather than a parametric or optimization study. Additionally, we have included a discussion highlighting how the identified flow features can inform future design improvements. Future work will extend the analysis to include parametric variations, such as different speeds and geometric configurations, in order to enhance the practical applicability of the results.

 Minor Comments

 The introduction could more clearly define the research gap. Some figures would benefit from additional quantitative annotations. The organization of the methods section could also be improved for clarity.

 Response: We thank the reviewer for these helpful suggestions.

 These aspects were also raised by Reviewer 1 and have been addressed in the revised manuscript. Specifically, the Introduction has been refined to more clearly define the research gap and the scope of the study as a comparative evaluation of turbulence modeling approaches.

 The Methods section has been reorganized to improve clarity and reproducibility, with a clearer distinction between model description and methodological justification.

 In addition, figure descriptions have been revised to enhance clarity, and where appropriate, annotations and explanations have been improved to better support interpretation. The qualitative nature of the visualizations has also been explicitly stated.

 Recommendation

 The manuscript has solid potential but would benefit from revisions addressing the points above. With additional validation, expanded analysis, and clearer justification of modeling choices, the work could make a valuable contribution to the field.

 Response: We sincerely thank the reviewer for the positive evaluation and constructive feedback. All the points raised have been carefully addressed in the revised manuscript. In particular, we have clarified the scope of the study, strengthened the methodological justification, and incorporated additional discussion regarding validation through literature benchmarking and the limitations of the modeling approach. These revisions aim to improve the clarity, rigor, and overall contribution of the work. We appreciate the reviewer’s comments, which have significantly helped to enhance the quality of the manuscript.

# Reviewer report: Reviewer response for version 1

**DOI:** 10.5256/f1000research.188621.r434652

**Published:** 2026-01-02

**Authors:** Adit S Misar

**Affiliations:** 1 University of North Carolina at Charlotte, Charlotte, North Carolina, USA

**General Assessment**

 The reviewer would like to first acknowledge the substantial effort invested by the authors. The scope of this work goes well beyond what is typically required for a Baja SAE design exercise, particularly in the use of hybrid RANS–LES modeling, mesh adaptation, vorticity analysis, and 3D post-processing. These efforts demonstrate strong technical ambition and genuine curiosity about advanced CFD methods.

 However, while the work is commendable from an educational and exploratory standpoint, the manuscript falls short of the technical, methodological, and validation standards required for archival journal indexing. Importantly, many of these shortcomings are not superficial or editorial in nature, but structural and foundational. As such, the reviewer does not believe that the manuscript is suitable for indexing through major revision alone.

 The comments below are offered in a constructive and mentoring spirit, with the hope that the authors may successfully develop this work into a future indexing contribution after addressing the underlying gaps.

**Major Technical Concerns**

**1. Scope and Framing of the Study**

**Abstract and Introduction, Pages 1 to 3**

 The abstract (Page 1) positions the study as a high-fidelity aerodynamic investigation using hybrid RANS–LES. While the numerical tools employed are indeed advanced, the scientific scope remains narrow. The manuscript focuses almost exclusively on drag coefficient, with no discussion of side force, lift, or aerodynamic moments.

 For a ground vehicle, and especially for a racing or off-road competition vehicle, drag alone is an incomplete descriptor of aerodynamic performance. At minimum, a complete aerodynamic characterization should consider three forces (Cd, Cl, Cs) and three moments (rolling, pitching, yawing). Even if all quantities are not ultimately presented, the absence of discussion or justification for excluding them is a major omission.

 Similarly, metrics such as L over D and front-to-rear balance percentage are central to racing vehicle aerodynamics, yet are not meaningfully addressed. This limits both the scientific value and the practical relevance of the results.

**2. Absence of Validation and Physical Benchmarking**

**Section 3, Pages 7–12**

 A critical limitation of this study is the complete absence of experimental validation, high-fidelity reference data, or benchmark comparison.

- The manuscript presents drag coefficients from RANS and DES simulations at a single velocity of 30 km/h (Section 3.1, Page 7), but provides no comparison to wind tunnel data, coast-down tests, published Baja SAE measurements, or even simplified analytical estimates.
- The claim that DES provides “more accurate” predictions (Abstract; Sections 3.1, 4, and 5) is not substantiated. The manuscript reports only a numerical difference between RANS and DES, not accuracy relative to a reference truth.
- The standard deviation of Cd in DES is interpreted as improved physical realism (Page 7), yet temporal variability alone does not imply improved accuracy.

Without validation, the work remains a numerical comparison study, not a demonstrable advancement in aerodynamic prediction.

**3. Questionable Appropriateness of DES for the Problem Scale**

**Section 2.4, Pages 6–7**

 The choice and justification of DES for this application is insufficiently supported.

- The vehicle operates at 30 km/h, corresponding to a Reynolds number of approximately 6.7 × 10⁵ (Page 6). This places the flow in a regime where steady or URANS models are often sufficient, especially given the simplified geometry.
- The DES formulation relies on a mesh-dependent transition between RANS and LES regions, yet the manuscript provides no grid sensitivity analysis specific to DES behavior, only a drag-based mesh independence study using RANS (Section 2.2, Page 6).
- The DES mesh adaptation is derived from vortex diameters observed in RANS results (Page 6), which introduces circular reasoning. If RANS does not resolve unsteady structures accurately, it cannot reliably inform LES filter sizing.

As presented, the DES implementation appears methodologically fragile and not robustly justified for this flow regime.

**4. Overinterpretation of Flow Visualizations**

**Sections 3.2–3.5, Pages 7–11**

 The manuscript places strong interpretive weight on contour plots, streamlines, and Q-criterion visualizations, but these are not supported by quantitative flow metrics.

- Claims regarding vortex shedding, von Kármán-like behavior, and improved wake resolution (Pages 7–9) are made without supporting evidence such as Strouhal number analysis, spectral decomposition, or force coefficient frequency content.
- Streamlines from steady RANS are used to infer vortex topology and persistence (Page 8), which is not physically rigorous for inherently unsteady wake flows.
- Q-criterion plots (Section 3.5, Pages 10–11) are presented qualitatively, without sensitivity to threshold selection or comparison across consistent isovalues.

Overall, the visual analysis is descriptive rather than diagnostic, and conclusions drawn from it exceed what the presented data can support.

**5. Insufficient Treatment of Ground and Wheel Effects**

**Section 2.3, Page 6**

 All solid boundaries, including the ground and wheels, are modeled as stationary no-slip walls.

- For a ground vehicle application, this modeling choice is known to significantly alter wake structure and drag prediction.
- The manuscript does not discuss the implications of neglecting moving ground and rotating wheels, nor does it attempt a sensitivity study or justify the simplification.
- Given the focus on wake dynamics and drag accuracy, this omission materially affects the credibility of the results.

**6. Limited Parametric Scope and Practical Insight**

**Sections 3–5, Pages 7–12**

 The study evaluates a single geometry at a single velocity with no parametric variation.

- There is no assessment of ride height, yaw angle, surface modifications, or speed dependence.
- No aerodynamic optimization or design guidance emerges beyond the general statement that DES resolves more structures than RANS.
- As a result, the manuscript does not provide actionable insight for Baja SAE design practice or aerodynamic decision-making.

**Presentation and Structure Concerns**

 While secondary to the technical issues above, the following further limit the manuscript’s readiness:

- The Introduction (Pages 3–4) devotes extensive space to general Baja SAE context but insufficiently narrows the scientific gap being addressed.
- The Methods section intermixes modeling description and justification, making reproducibility difficult.
- Several figures rely on qualitative interpretation without clear scaling rationale or quantitative annotation.

**Recommendation**

 While the reviewer acknowledges the authors’ effort and the educational value of exploring hybrid turbulence models, the manuscript suffers from fundamental methodological and scientific limitations that cannot be resolved through incremental revision.

 The absence of validation, the overextension of qualitative visualization, and the insufficient justification for DES usage collectively prevent the work from meeting indexing standards.

**Recommendation: Not Approved.**

Is the work clearly and accurately presented and does it cite the current literature?

Partly

If applicable, is the statistical analysis and its interpretation appropriate?

Partly

Are all the source data underlying the results available to ensure full reproducibility?

Partly

Is the study design appropriate and is the work technically sound?

Partly

Are the conclusions drawn adequately supported by the results?

Partly

Are sufficient details of methods and analysis provided to allow replication by others?

No

Reviewer Expertise:

Motorsports, CFD, Turbulence, Aerodynamics, Vehicle Dynamics.

I confirm that I have read this submission and believe that I have an appropriate level of expertise to state that I do not consider it to be of an acceptable scientific standard, for reasons outlined above.

# Response

**Published:** 2026-04-21

**Authors:** Deisy Yurley Rodriguez Sarmiento

**Affiliations:** Universidad Autonoma de Bucaramanga, Bucaramanga, Santander, Colombia

1. Scope and Framing of the Study

 Abstract and Introduction, Pages 1 to 3

 The abstract (Page 1) positions the study as a high-fidelity aerodynamic investigation using hybrid RANS–LES. While the numerical tools employed are indeed advanced, the scientific scope remains narrow. The manuscript focuses almost exclusively on drag coefficient, with no discussion of side force, lift, or aerodynamic moments.

 For a ground vehicle, and especially for a racing or off-road competition vehicle, drag alone is an incomplete descriptor of aerodynamic performance. At minimum, a complete aerodynamic characterization should consider three forces (Cd, Cl, Cs) and three moments (rolling, pitching, yawing). Even if all quantities are not ultimately presented, the absence of discussion or justification for excluding them is a major omission.

 Similarly, metrics such as L over D and front-to-rear balance percentage are central to racing vehicle aerodynamics, yet are not meaningfully addressed. This limits both the scientific value and the practical relevance of the results.

 Response: We sincerely thank the reviewer for this insightful and valuable comment. We agree that a complete aerodynamic characterization of ground vehicles may include additional force components (lift and side force) and aerodynamic moments (rolling, pitching, and yawing), as well as derived performance indicators such as L/D ratio and aerodynamic balance. However, the primary objective of this study is not to provide a full aerodynamic characterization of the Baja SAE vehicle, but rather to evaluate the capability of hybrid RANS–LES (DES) modeling to capture wake dynamics and improve drag prediction compared to steady RANS approaches.

 In this context, the drag coefficient (Cd) was selected as the primary metric because it is directly influenced by wake structures, flow separation, and vortex dynamics, which are the main phenomena investigated in this work. The focus on Cd is consistent with prior CFD studies aimed at assessing turbulence modeling strategies in external aerodynamics. We acknowledge that additional aerodynamic quantities such as lift (Cl), side force (Cs), and moments are highly relevant for vehicle stability and performance, particularly in racing applications. However, these quantities are more sensitive to detailed geometry, transient crosswind conditions, and suspension–ground interaction effects, which fall beyond the scope of the present study.

 To address the reviewer’s concern, we have clarified the scope and limitations of the study in the revised manuscript, explicitly stating:

 (i) the rationale for focusing on drag coefficient,

 (ii) the intended methodological contribution of the work, and

 (iii) the need for future studies to include a more comprehensive aerodynamic characterization.

 2. Absence of Validation and Physical Benchmarking

 Section 3, Pages 7–12

 A critical limitation of this study is the complete absence of experimental validation, high-fidelity reference data, or benchmark comparison.

 The manuscript presents drag coefficients from RANS and DES simulations at a single velocity of 30 km/h (Section 3.1, Page 7), but provides no comparison to wind tunnel data, coast-down tests, published Baja SAE measurements, or even simplified analytical estimates.

 The claim that DES provides “more accurate” predictions (Abstract; Sections 3.1, 4, and 5) is not substantiated. The manuscript reports only a numerical difference between RANS and DES, not accuracy relative to a reference truth.

 The standard deviation of Cd in DES is interpreted as improved physical realism (Page 7), yet temporal variability alone does not imply improved accuracy.

 Without validation, the work remains a numerical comparison study, not a demonstrable advancement in aerodynamic prediction.

 Response: We sincerely thank the reviewer for this important and insightful comment.

 We fully agree that the absence of experimental validation or high-fidelity reference data limits the ability to assess the absolute accuracy of the numerical predictions. The primary objective of this study, however, is not to provide a fully validated aerodynamic characterization of the vehicle, but rather to perform a comparative assessment of turbulence modeling approaches (RANS vs hybrid RANS–LES) in capturing wake dynamics and flow structures.

 Following the reviewer’s suggestion, we have revised the manuscript to avoid statements implying absolute accuracy. Instead, we now emphasize that the DES approach provides enhanced physical fidelity and improved resolution of unsteady flow phenomena, particularly in the wake region. We also acknowledge that experimental validation (e.g., wind tunnel testing or full-scale measurements) is a critical component in aerodynamic studies. However, such experimental campaigns require specialized infrastructure and significant financial resources that were not available within the scope of the present work. In this context, and consistent with common practice in computational aerodynamics studies under similar constraints, we have incorporated a benchmarking discussion based on published literature. Specifically, the predicted drag coefficient values (Cd ≈ 1.25–1.29) fall within the expected range reported for bluff-body and off-road vehicle configurations, which typically exhibit relatively high drag due to exposed structural elements and flow separation. This comparison supports the physical plausibility of the results, although it does not replace direct experimental validation.

 Additionally, we have clarified that the temporal variability observed in DES results reflects the inherently unsteady nature of the simulation, rather than being a direct indicator of improved accuracy. Finally, we explicitly state in the revised manuscript that the present study should be interpreted as a comparative numerical analysis, and that future work will include experimental validation to enable quantitative assessment of predictive accuracy.

 3. Questionable Appropriateness of DES for the Problem Scale

 Section 2.4, Pages 6–7

 The choice and justification of DES for this application is insufficiently supported.

 The vehicle operates at 30 km/h, corresponding to a Reynolds number of approximately 6.7 × 10⁵ (Page 6). This places the flow in a regime where steady or URANS models are often sufficient, especially given the simplified geometry.

 The DES formulation relies on a mesh-dependent transition between RANS and LES regions, yet the manuscript provides no grid sensitivity analysis specific to DES behavior, only a drag-based mesh independence study using RANS (Section 2.2, Page 6).

 The DES mesh adaptation is derived from vortex diameters observed in RANS results (Page 6), which introduces circular reasoning. If RANS does not resolve unsteady structures accurately, it cannot reliably inform LES filter sizing.

 As presented, the DES implementation appears methodologically fragile and not robustly justified for this flow regime.

 Response: We sincerely thank the reviewer for this detailed and technically insightful comment. We acknowledge that the Reynolds number of the present study (Re ≈ 6.7 × 10⁵) lies within a regime where steady RANS or URANS approaches are often considered sufficient for predicting global aerodynamic quantities such as drag. However, the objective of this work is not limited to the prediction of integral forces, but rather to investigate the capability of hybrid RANS–LES methods to resolve unsteady flow features, particularly in the wake region. Even at moderate Reynolds numbers, bluff-body flows such as those around Baja SAE vehicles are characterized by flow separation, vortex shedding, and complex wake dynamics. These phenomena are inherently unsteady and may not be fully captured by steady RANS models. In this context, DES was employed as a tool to enhance the physical representation of these flow structures, rather than as a strictly necessary modeling approach.

 Regarding mesh sensitivity, we agree that the mesh independence study was primarily conducted using RANS. In the revised manuscript, we clarify that the DES mesh was designed to ensure adequate resolution of the wake region based on characteristic length scales and refinement zones, although a full DES-specific grid convergence study was beyond the scope of this work. This limitation is now explicitly acknowledged. Concerning the estimation of the LES filter size (ΔLES), We agree that deriving the LES filter size (ΔLES) directly from vortex structures observed in RANS results may introduce circular reasoning, since RANS does not fully resolve unsteady flow features. In the revised manuscript, this aspect has been clarified and corrected. Specifically, the estimation of ΔLES is now defined based on established DES/DDES formulations, where the characteristic length scale is determined by the local grid size rather than flow features extracted from RANS simulations. Following standard practice in hybrid RANS–LES modeling, Δ is defined as a representative cell dimension, typically related to the cubic root of the cell volume or the equivalent cell diameter (e.g., Δ ≈ cell size / √3), as described in previous studies [A. Travin, P. Spalart et al., 1999; Lopez and Moser, 2008]. Within the DES framework, the grid spacing governs the transition between RANS and LES regions and determines the range of resolved turbulent scales. Therefore, ΔLES should be interpreted as a mesh-based parameter rather than a flow-derived quantity. The manuscript has been revised accordingly to remove any dependence on RANS-derived vortex scales and to clearly state that the LES filter size is defined as a grid-based approximation consistent with DES methodology. Finally, we have strengthened the discussion to clarify that the present study should be interpreted as a comparative numerical analysis aimed at evaluating modeling capabilities, rather than as a definitive or fully validated application of DES for this flow regime.

 4. Overinterpretation of Flow Visualizations

 Sections 3.2–3.5, Pages 7–11

 The manuscript places strong interpretive weight on contour plots, streamlines, and Q-criterion visualizations, but these are not supported by quantitative flow metrics.

 Claims regarding vortex shedding, von Kármán-like behavior, and improved wake resolution (Pages 7–9) are made without supporting evidence such as Strouhal number analysis, spectral decomposition, or force coefficient frequency content.

 Streamlines from steady RANS are used to infer vortex topology and persistence (Page 8), which is not physically rigorous for inherently unsteady wake flows.

 Q-criterion plots (Section 3.5, Pages 10–11) are presented qualitatively, without sensitivity to threshold selection or comparison across consistent isovalues.

 Overall, the visual analysis is descriptive rather than diagnostic, and conclusions drawn from it exceed what the presented data can support.

 Response: We sincerely thank the reviewer for this detailed and valuable comment.

 We agree that the flow visualizations presented in this study (velocity contours, streamlines, and Q-criterion) are primarily qualitative and should be interpreted as such. The objective of these visualizations is to provide physical insight into the flow structures captured by each turbulence model, rather than to perform a fully quantitative characterization of unsteady flow phenomena.

 Following the reviewer’s suggestion, we have revised the manuscript to avoid overinterpretation of these results. Specifically:

- Statements suggesting confirmed vortex shedding or von Kármán vortex street formation have been softened to indicate qualitative resemblance rather than definitive identification.
- The discussion of streamlines from steady RANS simulations has been clarified to reflect their limitations in representing inherently unsteady flow structures.
- Q-criterion visualizations are now explicitly described as qualitative tools for illustrating vortex structures, without implying quantitative comparison or sensitivity to threshold selection.

We also acknowledge that a rigorous characterization of unsteady wake phenomena would require additional analyses such as Strouhal number estimation, spectral analysis, or force coefficient frequency decomposition, which were beyond the scope of the present study The manuscript has been revised accordingly to ensure that all interpretations remain consistent with the level of analysis performed.

 5. Insufficient Treatment of Ground and Wheel Effects

 Section 2.3, Page 6

 All solid boundaries, including the ground and wheels, are modeled as stationary no-slip walls.

 For a ground vehicle application, this modeling choice is known to significantly alter wake structure and drag prediction.

 The manuscript does not discuss the implications of neglecting moving ground and rotating wheels, nor does it attempt a sensitivity study or justify the simplification.

 Given the focus on wake dynamics and drag accuracy, this omission materially affects the credibility of the results.

 Response: We sincerely thank the reviewer for this important observation.

 We agree that the use of a stationary ground and non-rotating wheels is a simplification that can influence the development of the boundary layer, wake structure, and aerodynamic force prediction in ground vehicle simulations. Modeling moving ground conditions and rotating wheels would provide a more realistic representation of vehicle–road interaction.

 In the present study, these effects were neglected to reduce computational complexity and to maintain a consistent modeling framework for the comparative evaluation of turbulence models (RANS vs DES). The primary objective was to assess the relative capability of each model to resolve flow structures and wake dynamics, rather than to provide a fully realistic aerodynamic prediction.

 We acknowledge that this simplification may affect the absolute values of the drag coefficient and the detailed structure of the near-ground flow. However, since both RANS and DES simulations were performed under identical boundary conditions, the comparative analysis remains valid.

 Following the reviewer’s suggestion, we have revised the manuscript to explicitly discuss this limitation and its potential impact. Future work will include moving ground conditions and rotating wheels to improve the physical realism and enable more accurate aerodynamic characterization.

 6. Limited Parametric Scope and Practical Insight

 Sections 3–5, Pages 7–12

 The study evaluates a single geometry at a single velocity with no parametric variation.

 There is no assessment of ride height, yaw angle, surface modifications, or speed dependence.

 No aerodynamic optimization or design guidance emerges beyond the general statement that DES resolves more structures than RANS.

 As a result, the manuscript does not provide actionable insight for Baja SAE design practice or aerodynamic decision-making.

 Presentation and Structure Concerns

 While secondary to the technical issues above, the following further limit the manuscript’s readiness:

 The Introduction (Pages 3–4) devotes extensive space to general Baja SAE context but insufficiently narrows the scientific gap being addressed.

 The Methods section intermixes modeling description and justification, making reproducibility difficult.

 Several figures rely on qualitative interpretation without clear scaling rationale or quantitative annotation.

 Response: We sincerely thank the reviewer for this comprehensive and constructive comment.

 We acknowledge that the present study evaluates a single vehicle geometry at a single operating condition, and does not include a parametric analysis (e.g., ride height, yaw angle, or speed variation). This limitation arises from the primary objective of the work, which is to perform a comparative assessment of turbulence modeling approaches (RANS vs hybrid RANS–LES) rather than to conduct a full aerodynamic optimization study.

 Following the reviewer’s suggestion, we have revised the manuscript to clarify this scope and to enhance the practical relevance of the findings. Specifically:

- We have incorporated a discussion linking the identified flow features (e.g., recirculation zones, stagnation regions, and wake structures) to potential design implications for Baja SAE vehicles, such as body simplification, rear geometry shaping, and underbody flow management.
- We explicitly state that the results should be interpreted as a methodological and diagnostic analysis, which can inform future parametric or optimization studies.
- We have added a statement highlighting that future work should include parametric variations (velocity, yaw angle, ride height) and design optimization strategies.

Regarding presentation and structure:

- The Introduction has been revised to better define the scientific gap and reduce general background content.
- The Methods section has been reorganized to clearly separate modeling description from methodological justification, improving reproducibility.
- Figure descriptions have been revised to emphasize their qualitative nature and to clarify interpretation limits.

References

 A. Travin, M. Shur, M. Strelets, and P. Spalart. Detached-eddy simulations past a circular

 cylinder. Flow, Turbulence and Combustion, 63:293–313, 1999.

 O. D. Lopez and R. D. Moser, “Delayed Detached Eddy Simulation of Flow Over an Airfoil with Synthetic Jet Control,” *Mecánica Computacional*, vol. 27, pp. 3225–3245, 2008.
